# The Flagellin FliC of *Clostridium difficile* Is Responsible for Pleiotropic Gene Regulation during *In Vivo* Infection

**DOI:** 10.1371/journal.pone.0096876

**Published:** 2014-05-19

**Authors:** Amira Barketi-Klai, Marc Monot, Sandra Hoys, Sylvie Lambert-Bordes, Sarah A. Kuehne, Nigel Minton, Anne Collignon, Bruno Dupuy, Imad Kansau

**Affiliations:** 1 Faculté de Pharmacie, EA4043, Université Paris Sud, Châtenay-Malabry, France; 2 Laboratoire Pathogenèse des Bactéries Anaérobies, Institut Pasteur, Paris, France; 3 Clostridia Research Group, Centre for Biomolecular Sciences, School of Life Sciences, University of Nottingham, Nottingham, United Kingdom; Institude Pasteur, France

## Abstract

*Clostridium difficile* is the main agent responsible for hospital acquired antibiotic associated diarrhoea. In recent years, epidemic strains have emerged causing more severe infections. Whilst *C. difficile* has two major virulence factors, toxins TcdA and TcdB, it is generally accepted that other virulence components of the bacterium contribute to disease. Previously, it has been suggested that flagella expression from pathogenic bacteria might be implicated in virulence. In a recent study, we observed an increased mortality in a gnotobiotic mouse model when animals were colonized with an isogenic *fliC* mutant constructed in the PCR-ribotype 027 (B1/NAP1) strain R20291, while animals survived when colonized by the parental strain or after colonization by other high-toxin-producing *C. difficile* strains. To understand the reasons for this increased virulence, we compared the global gene expression profiles between the *fliC*-R20291 mutant and its parental strain using an *in vitro* and *in vivo* transcriptomic approach. The latter made use of the gnotobiotic mouse model. Interestingly, in the *fliC* mutant, we observed considerable up-regulation of genes involved in mobility, membrane transport systems (PTS, ABC transporters), carbon metabolism, known virulence factors and sporulation. A smaller but significant up-regulation of genes involved in cell growth, fermentation, metabolism, stress and antibiotic resistance was also apparent. All of these genes may be associated with the increased virulence of the *fliC*-R20921 mutant. We confirmed that the *fliC* mutation is solely responsible for the observed changes in gene expression in the mutant strain since expression profiles were restored to that of the wild-type strain in the *fliC*-complemented strain. Thus, the absence of FliC is directly or indirectly involved in the high mortality observed in the *fliC* mutant infected animals. Therefore, we provide the first evidence that when the major structural component of the flagellum is neutralized, deregulation of gene expression can occur during infection.

## Introduction

*Clostridium difficile* is the main agent responsible for hospital acquired antibiotic associated diarrhoea in North America and Europe. Since the last decade, the morbidity and mortality rates of nosocomial *C. difficile* infection (CDI) continue to rise, particularly following the emergence of epidemic *C. difficile* strains (027/BI/NAP1) [1], [2]. However, other *C. difficile* strains belonging to different PCR ribotypes (PR) have also emerged and appear to be as virulent as the PR 027 strains depending on geographical location. For example, 053, 106, 001, 018 and 078 PR strains are considered as dominant in Austria (053), England (106), Germany (001) and Italy (018, 078) [3]–[7]. In the Netherlands, the prevalence of PR 078 strains increased from 3% to 13% between 2005 and 2008 [8]. A recent study conducted in 16 countries of Americas, Europe and Australia, shows that PR 027 strains are the most prevalent strains in North America whereas PR 001 strains are the most common in Europe [9].

*C. difficile* colonizes the gut of humans and animals. Disease symptoms range from self-limiting diarrhoea to fatal pseudomembranous colitis. It is well recognised that the main virulence factors of *C. difficile* are the clostridial toxins TcdA and TcdB [10]–[14]. However, other factors certainly contribute to gut colonization and development of intestinal lesions. *C. difficile* expresses multiple putative surface adhesins and colonization factors, including cell surface-associated proteins (S-layer and SLPs), fibronectin-binding protein FbpA, proteases such as Cwp84, hydrolytic enzymes, heat-shock proteins GroEl [15]–[17] and flagellar cap FliD and flagellin FliC structural components [18].

For most pathogens, flagella are recognised as essential virulence factors. Their role in motility and chemotaxis increases the occurrence of potential interactions between the pathogen and the epithelial mucosal surface. Moreover, bacterial flagella are involved in infection through their roles in host-cell adhesion, cell invasion, auto-agglutination and formation of biofilms, as well as in the regulation and secretion of non-flagellar bacterial proteins involved in the virulence process. Specifically, the participation of flagella has been demonstrate in the adherence and colonization of mucosal membranes has been shown in *Pseudomonas aeruginosa* [19], [20], *Helicobacter pylori* [21], *Aeromonas caviae* [22] and *Campylobacter jejuni* [23], in the internalization of bacteria into epithelial cells in *C. jejuni* [24], [25] and *Legionella pneumophila* [24] and in biofilm formation by *A. caviae* [26], *Bacillus subtilis* [27] and *C. difficile* [28].

The involvement of flagella in other important cellular processes has also been shown. For example, the flagellar apparatus of *C. jejuni* is responsible for the secretion of non-flagellar proteins involved in cell adhesion and internalization of the bacteria [29]–[31]. In addition, the flagellar motor structure of some bacteria plays an important role in regulating flagellar assembly via different sigma factors (σ) and in regulating the expression of non-flagellar genes through their function as sensors of environmental conditions [32].

Early indications that flagella were important in the virulence of *C. difficile* was derived from the observation that flagellated, motile *C. difficile* attach more efficiently to the caecal wall of axenic mice than non-flagellated strains of the same serogroup [33]. Moreover, flagellin and the flagellar cap were later implicated as one of the multiple cell-surface adhesins of the bacterium [18]. More definitive evidence that flagella are involved in virulence was subsequently made possible by the development of gene tools that allowed the generation of directed mutants [34]. Thus, Dingle *et al*. [35] were able to construct isogenic mutants of *C. difficile* strain 630Δ*erm* in which either *fliC* and *fliD* had been inactivated. These flagella mutants adhered more strongly to Caco2 cells *in vitro* and showed increased toxicity *in vitro* and virulence *in vivo*, the latter as tested in the hamster model of infection. However, given the high degree of variation of flagella-related gene content amongst *C. difficile* strains [36], [37], it was of value to investigate the role of flagella in more relevant epidemic strains.

We recently compared the effect of flagella and flagellum-mediated motility between the epidemic *C. difficile* UK outbreak strain R20291 and non-epidemic strain 630Δ*erm* [38]. This study showed significant differences between the two *C. difficile* strains in terms of adherence to epithelial cells, toxicity and virulence *in vivo*. Competition studies in the gnotobiotic mouse model (dixenic mice) demonstrated that an isogenic *fliC* mutant in strain R20291 exhibited a reduced ability to colonize compared to the wild-type strain, suggesting that flagella have a role in the intestinal colonization of mice [38]. Surprisingly, when mice were infected with the *fliC*-R20291 mutant alone (monoxenic mice), about 70% of animals died 48 h post-infection. Such a high mortality rate has never been observed previously in this monoxenic mouse model, as these mice generally survive even after colonization by other high-toxin-producing *C. difficile* strains. These unexpected observations may suggest that virulence traits unrelated to the overexpression of toxins are expressed *in vivo* in the absence of the *C. difficile* flagellin FliC.

In the present work we compared the global gene expression profiles between the *fliC* mutant of *C. difficile* R20291 and its parental strain during *in vivo* infection. This comparative transcriptomic study shows that by disabling the structural component FliC of the flagellum of *C. difficile* R20291, more than 300 genes which are distributed in several functional categories, are differentially expressed *in vivo*. Thus, by regulating the expression of flagellar and non-flagellar genes, FliC could be involved in the virulence of *C. difficile* R20291.

## Results and Discussion

In a previous work [38], we constructed a *fliC* mutant of the epidemic PCR-ribotype 027 *C. difficile* strain R20291 and tested it's *in vitro* and *in vivo* colonization properties. Unexpectedly, the majority of those mice (70%) infected with the *fliC* mutant strain presented symptoms of CDI and succumbed within a few days post-infection. In contrast, mice infected with the wild-type strain survived. A similar observation was reported by Dingle et al. who showed that a *fliC* mutant in strain 630Δ*erm* was more virulent than its parental strain in hamsters by overproduction of toxins, indicating that suppression of motility may be a pathogenic strategy employed by *C. difficile* during infection in hamsters [35].

### Absence of FliC is associated to *in vivo* differential gene expression in *C. difficile*

The high mortality rate of monoxenic mice when infected with the *C. difficile fliC* mutant in R20291 [38] led us to conduct an *in vivo* transcriptomic analysis to compare gene expression differences between the R20291 *fliC* mutant and parental strain. Groups of germ-free mice were orally challenged with either *C. difficile* R20291 or the *fliC* mutant. Bacterial RNA was recovered from the caeca at 14 h post-infection, transcribed into cDNA and then labeled for hybridization to DNA microarrays (Agilent™) as described in the Materials and Methods.

**Figure 1:**
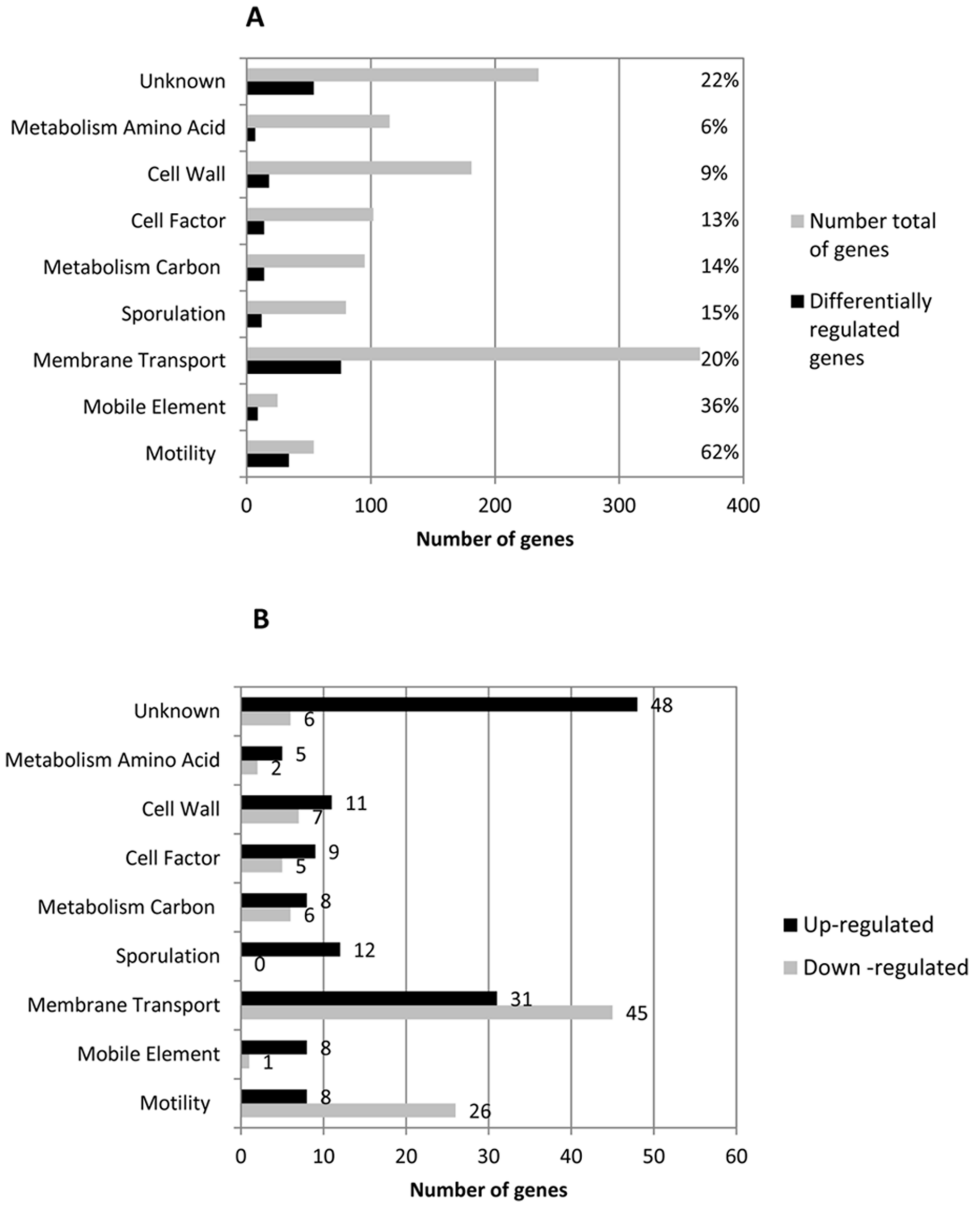
Functional clusters of *in vivo* differentially expressed genes. Differentially expressed genes in the *fliC* mutant compared to wild-type R20291 from caeca of 14 h post-infection mice were classified in functional groups according to their involvement in the biological process categories. (**A**) The number of analyzed genes is represented in green bars and the number of significantly differentially expressed genes is shown in black bars. Percentages of differentially regulated genes are indicated at right. (**B**) The numbers of up- and down-regulated genes for each cluster are indicated in green and black bars, respectively.

When we compared the *in vivo* transcriptomic profiles of the R20291 *C. difficile* strain to its isogenic *fliC* mutant, we observed a total of 310 genes differentially expressed at 14 h post-infection. In order to better analyze genes differentially expressed between the two strains, regulated genes were assigned to functional categories (Figure 1A). Distribution of up- and down-regulated genes (179 and 131, respectively) is shown in Fig. 1B. Whereas the majority of genes differentially expressed were up-regulated we observed an exception for the motility and membrane transport gene clusters, which were mainly down-regulated (26 out of 34 (n = 54) and 45 out of 76 (n = 365), respectively; Fig. 1B). Interestingly, the highest gene expression differences were observed for genes involved in motility (62%, n = 54), membrane transport systems (PTS and ABC transporters; 20%, n = 365), sporulation (15%, n = 80) and carbon metabolism (14%, n = 95), suggesting a co-regulation between expression of flagella genes and genes whose products are involved in the early stages of intestinal colonization and bacterial virulence (Figure 1A).

### Absence of the *C. difficile* FliC is associated to *in vitro* differential gene expression, which is restored by trans *fliC* complementation

To confirm that the differences seen were a consequence of the inactivation of *fliC*, complementation studies were undertaken. The *fliC* structural gene was cloned into the complementation plasmid pMTL84151 (Materials and Methods) and the resultant recombinant plasmid shown to restore the swimming and swarming phenotype of the mutant to those of the wild-type. In parallel experiments, the presence of the vector-only control, pMTL84151, was shown to have no effect on the growth rate of either the wild-type strain or the *fliC* mutant (Figure S1). Maintenance of the complementation plasmid in the mutant strain requires the imposition of antibiotic selection. This is not possible during the *in vivo* infection process. As an alternative, the transcriptome profiles of the wild-type R20291 strain, its isogenic *fliC* mutant and the trans *fliC*-complemented strain (see below) were compared at the onset of the stationary phase (14 h growth after inoculation) of cells grown in peptone yeast (PY) media.

The *in vitro* comparative transcriptome analysis showed that 258 genes were differentially expressed in the *fliC* mutant compared to the wild-type strain, whilst no difference in gene expression was observed when the trans *fliC*-complemented strain was compared to the wild-type strain. Thus, we can conclude that the absence of FliC alone is responsible for the differential gene expression observed *in vitro* in the *fliC* mutant and by extrapolation in the *in vivo* experiment. It is interesting to note that among those genes differentially regulated in the *fliC* mutant, only 36 were common to both the *in vitro* and *in vivo* analyses (see below).

### Validation of *in vivo* microarray data using qRT-PCR

To validate the transcriptomic profile data, we selected two sets of 24 genes differentially expressed *in vivo* and six house-keeping genes (*dna polIII*, *rrs* -*16S*-, *rpoA*, *gyrA*, *gluD* and *rpsJ*), and performed qRT-PCR on RNA extracted from caecal bacteria (Table S2 in File S1). RT-qPCR results and microarray data exhibited high correlation coefficients (R^2^: 0.87; Table S2 in File S1). However, these two technologies revealed a quantitative difference in the fold changes, which has also been reported by others [35]. This indicates that the fold change in gene expression presented in the microarray analysis is probably underestimated.

### Functional clusters of *in vivo* differentially expressed genes

As indicated, all genes differentially expressed in the *fliC* mutant compared to the wild-type were distributed in several functional categories. We have focused our interest on functional gene clusters that could be related to the high virulence of the *fliC* mutant observed in the monoxenic mouse model.

### Flagellar genes

Compared to the parental strain, disruption of the *fliC* gene in *C. difficile* R20291 leads to a non-flagellated non-motile phenotype and an unexpected increased mortality of monoxenic mice [38]. Thus, we first analyzed the impact of the *fliC* inactivation on the expression of the flagellar locus and then on the other non-flagellar genes as previously described for the pathogen *C. jejuni* [30].

The major flagella regulon of *C. difficile* is grouped in several regions within the genome. The first region (F1) carries late stage flagellar genes, including the flagellar filament encoding gene *fliC*. Genes of the second region (F2), downstream of F1, encode flagellar glycan biosynthesis pathways, whereas genes of the last region (F3) encode early stage genes involved in flagellar hook and basal body biogenesis and the SigD transcriptional regulator of the F1 locus [39], [40]. The transcriptomic analysis shows that most genes from the F1 locus were up-regulated in the *fliC* mutant; among them *fliD*, encoding the flagellar capping protein gene FliD, which was 5-fold up-regulated as previously observed for a *fliC* mutant in *C. difficile* 630Δ*erm* [35]. In contrast, *flgM* (encoding a negative regulator of flagellin synthesis) and *flgK* (encoding a putative flagellar hook-associated protein) were down-regulated (Table 1). Genes belonging to the F3 locus, were generally unaffected, or in some cases slightly down-regulated in the *fliC* mutant. Overall these data indicate that the structural flagellar protein FliC plays an important role in co-regulating biosynthesis of *C. difficile* flagella. In support of this idea, Aubry *et al.* [39], suggested that the F3 locus genes were co-transcribed from a common promoter under the control of the vegetative sigma factor σ^A^. This was recently confirmed when a SigA-like consensus sequence was identified by RACE-PCR upstream of the start codon of *flgB*, the first gene of the F3 locus [41]. Moreover, *sigD* inactivation in *C. difficile* only slightly affects the expression of genes encoding the early flagellar genes [41], suggesting that their expression is partly independent of the expression of SigD. In contrast to the early-stage flagellar genes, SigD-dependent promoters were found upstream of several late-stage flagellar genes (F1 locus) whose expression was substantially decreased in the *sigD* mutant [39], [41]. In *B. subtilis*, the SigD-dependent transcription of late flagellar genes is repressed during initiation of flagella biosynthesis by FlgM, the anti-SigD factor, which prevents the association of SigD with the RNA polymerase. Further, FlgM is secreted from the cells through the assembled flagellar motor structure encoded by the F3 locus genes, releases SigD and enables SigD-dependent genes transcription [42]. Thus, the impact of the *fliC* mutation on *flgM* expression (4-fold down-regulated), in addition to the absence of filament structural gene that keep the flagella structure incomplete, could favor secretion of FlgM and explain, in part, the up-regulation of F3 locus genes in the *fliC* mutant.

**Table 1:** Flagellar genes differentially expressed *in vivo* in the *fliC* mutant.

Gene ID	Gene ortholog	Name	Description	Microarray fold change
CDR0227	CD630_02260*		Putative lytic transglycosylase	2.03
CDR0230	CD630_02290*	*flgM*	Negative regulator of flagellin synthesis (anti-sigma-d factor)	0.20
CDR0231	CD630_02300*		Putative flagellar biosynthesis protein	3.30
CDR0232	CD630_02310*	*flgK*	Flagellar hook-associated protein FlgK (or HAP1)	0.40
CDR0234	CD630_02330*	*fliW*	Flagella assembly factor FliW	2.03
CDR0235	CD630_02340*	*csrA*	Carbon storage regulator homolog CsrA	4.36
CDR0236	CD630_02350*	*fliS1*	Flagellar protein FliS	3.99
CDR0237	CD630_02360*	*fliS2*	Flagellar protein FliS2	2.17
CDR0238	CD630_02370*	*fliD*	Flagellar cap protein	5.05
CDR0239	CD196_0252		Conserved hypothetical protein	4.99
CDR0240	CD630_02390*	*fliC*	Flagellin subunit FliC	0.11
CDR0248	CD630_02450**	*flgB*	Flagellar basal-body rod protein FlgB	0.34
CDR0249	CD630_02460**	*flgC*	Flagellar basal-body rod protein FlgC	0.31
CDR0250	CD630_02470**	*fliE*	Flagellar hook-basal body complex protein FliE	0.25
CDR0251	CD630_02480**	*fliF*	Flagellar M-ring protein FliF	0.28
CDR0252	CD630_02490**	*fliG*	Flagellar motor switch protein FliG	0.18
CDR0253	CD630_02500**	*fliH*	Flagellar assembly protein FliH	0.35
CDR0254	CD630_02510**	*fliI*	Flagellum-specific ATP synthase subunit beta FliI	0.39
CDR0255	CD630_02520**	*fliJ*	Flagellar protein FliJ	0.41
CDR0256	CD630_02530**	*fliK*	Flagellar hook-length control protein FliK	0.30
CDR0257	CD630_02540**	*flgD*	Basal-body rod modification protein FlgD	0.29
CDR0258	CD630_02550**	*flgE*	Flagellar hook protein FlgE (distal rod protein)	0.32
CDR0259	CD630_02551**	*flbD*	Flagellar protein FlbD	0.24
CDR0260	CD630_02560**	*motA*	Flagellar motor rotation protein MotA (chemotaxis protein)	0.19
CDR0261	CD630_02570**	*motB*	Flagellar motor rotation protein MotB (chemotaxis protein)	0.36
CDR0262	CD630_02580**	*fliL*	Flagellar basal body-associated protein FliL	0.51
CDR0264	CD630_02600**	*flip*	Flagellar biosynthesis protein FliP	0.40
CDR0267	CD630_02630**	*flhA*	Flagellar biosynthesis protein FlhA	0.41
CDR0268	CD630_02640**	*flhF*	Flagellar biosynthesis regulator FlhF (flagella-associated GTP-binding protein)	0.43
CDR0269	CD630_02650**	*flhG*	Flagellar number regulator FlhG	0.47
CDR0270	CD630_02660**	*fliA*	RNA polymerase sigma 28 factor for flagellar operon	0.42
CDR0271	CD630_02670**		Putative flagellar protein	0.31
CDR0274	CD630_02700**	*fliM*	Flagellar motor switch phosphatase FliM	0.45
CDR0276	CD630_02720**		Conserved hypothetical protein	0.47

*From F1 region (late-stage flagellar genes) and **F3 region (early-stage flagellar genes) of the flagellar operon from *C. difficile* 630 [39].

### Membrane transport

ABC type transporters, also called “traffic ATPases” are a set of phylogenetically related transport systems that are remarkably well conserved in their organization. These carriers play a role in the import or export of a considerable number of substrates, ranging from ions to macromolecules, and in many pathogens play a role in physiological functions that include nutrient transport, signal transduction, export of virulence factors and antibiotic resistance [43], [44]. Within the family of ABC transporters, peptide import systems, commonly referred to as Oligo-peptide permeases (Opp), are frequently involved in cell growth and nutrition. Indeed, Opp systems are involved in the internalization of peptides that are perceived by the cell as signals from the environment, or in intercellular communication including conjugation, sporulation, quorum sensing, etc. [43].

Eight genes encoding ATP binding cassette (ABC) transporter products were up- and down-regulated in the *fliC* mutant compared to the wild-type strain (Table 2). Among them, the operon *oppBCADF* (CDR0783-87), encoding an oligo-peptide-permease, was strongly down-regulated (16-fold) in the *fliC* mutant. This suggest that FliC could be involved at least indirectly in Opp functions such as the internalization of environmental signalling peptides and intercellular communication [43]. In contrast, three ABC transporter genes (CDR0551, CDR2981 and CDR2983), whose role is unclear, were up-regulated in the *fliC* mutant (Table 2). Interestingly, these genes are specific to 027 strains and their up-regulation could be involved in the “hypervirulence” of the *fliC* mutant strain. Overall, these observations may indicate that the differential expression of these particular ABC transporters could play a role in the virulence of *C. difficile* by improving the import or export of essential molecules involved in adaptive features of the bacterial cell and therefore in bacterial virulence.

**Table 2:** Membrane transport genes highly differentially expressed *in vivo* in the *fliC* mutant compared to strain R20291.

Gene ID	Gene ortholog	Name	Description	Microarray fold change
**ABC-type transport system genes**				
CDR0783	CD630_08530	*oppB*	ABC-type transport system, oligopeptide-family permease	0.07
CDR0784	CD630_08540	*oppC*	ABC-type transport system, oligopeptide-family permease	0.08
CDR0785	CD630_08550	*oppA*	ABC-type transport system, oligopeptide-family extracellular solute-binding protein	0.06
CDR0786	CD630_08560	*oppD*	ABC-type transport system, ATP-binding component	0.10
CDR0787	CD196_0806	*oppF*	Oligopeptide ABC transporter, ATP-binding protein	0.08
CDR0551	CD196_0569		ABC transporter, ATP-binding/permease protein	3.73
CDR2981	CD196_2934		Spermidine/putrescine ABC transporter ATP-binding subunit	3.39
CDR2983	CD196_2936		Putative ABC-type transport system, periplasmic component-like protein precursor	2.80
**PTS system genes**				
CDR2927	CD630_30880		Putative cellobiose-phosphate degrading protein	51.25
CDR2928	CD630_30890		PTS system, IIABC component	32.82
CDR3136	CD630_32750		Putative phosphosugar isomerase	10.06
CDR3137	CD630_32760		PTS system, mannose/fructose/sorbose IID component	4.44
CDR3138	CD630_32770		PTS system, mannose/fructose/sorbose IIC component	11.76
CDR3139	CD630_32780		PTS system, mannose/fructose/sorbose IIA component	10.83
CDR3140	CD630_32790		PTS system, mannose/fructose/sorbose IIB component	7.75
CDR2862	CD630_30270		PTS system, glucose-specific IIA component	0.07
CDR2863	CD630_30280		Putative phosphosugar isomerase	0.06
CDR2864	CD630_30290	*malY*	Bifunctional protein: cystathionine beta-lyase/repressor	0.05
CDR2865	CD630_30300	*malX*	PTS system, glucose-like IIBC component	0.04
CDR2866	CD630_30310		Transcription antiterminator, PTS operon regulator	0.37

The phosphoenolpyruvate-dependent phosphotransferase system (PTS) identified in many Gram negative and Gram positive bacteria is a complex enzyme system allowing transport of external sugars concomitant with its phosphorylation before release into the cell. The PTS is composed of enzyme I and a histidine-containing phosphocarrier protein (HPr), present in the cytoplasm, and the carbohydrate specific transporter enzyme II, consisting usually of three domains (EIIA, EIIB and EIIC). The enzymes EI and HPr allow phosphorylation of different sugars entering the cell through specific permeases [45]. Transport of rapidly metabolizable sugars (such as glucose) via the PTS can exert a significant influence on cell physiology such as regulation of many genes involved in the metabolism of other carbon sources, as well as on the control of transcriptional regulators including those of virulence genes [46]. Moreover, it has been demonstrated in *C. difficile* that glucose, and other rapidly metabolizable sugars, negatively regulate the expression of *tcdB* and *tcdA* [47] via the catabolic repression regulator CcpA (Catabolite control protein A) [48]. *C. difficile* has a considerable number of genes dedicated to carbohydrate transport and metabolism. Among them, we observed that expression of genes encoding specific permeases were up- and down-regulated in the *fliC* mutant compared to the parental strain (Table 2). Indeed, genes CDR2927-28, encoding a putative cellobiose-phosphate degrading protein and a glucose-like permease component (IIBC), were highly expressed (50- and 30-fold, respectively - Table 2). We noted that these genes are repressed by glucose in a CcpA-independent manner [49]. Interestingly, genes encoding components of a mannose/fructose/sorbose PTS (CDR3136-40) were 4- to 12-fold up-regulated, whereas genes CDR2862-2865, encoding compounds of a glucose-maltose PTS, were strongly down-regulated (14 to 25 fold - Table 2) in the *fliC* mutant. However, none of these genes seem to be induced or repressed by glucose or to be under the control of the pleiotropic regulator CcpA [49], suggesting that another carbon regulator system could be induced in the *fliC* mutant during *in vivo* infection.

In a general way, all the above transport systems play a crucial role in the defense of bacteria against toxic components as well as in their adaptation to different ecosystems [50]. Thus, we can hypothesize that these systems, whose regulation is connected directly or indirectly with the flagella synthesis, may be involved in the survival and/or adaptation of *C. difficile* to the gastro-intestinal environment.

### Sporulation genes

Spores are involved in host colonization and persistence of bacteria in the environment. In all spore forming bacteria, the first major morphological manifestation of sporulation is an asymmetric division (Stage II) that divides the sporulating cell into a larger mother cell and a smaller forespore (the future spore). Following septum formation, the mother cell engulfs the forespore (Stage III and IV). At later stages (Stage V and VI), a layer of peptidoglycan known as the spore cortex, is deposited between the inner and outer forespore membranes, and this layer is further encased within a protective proteinaceous coat followed by the release of the mature spore [51]. The initiation of sporulation is absolutely dependent on the phosphorylation of the transcription factor Spo0A. This master regulator of sporulation is phosphorylated in response to environmental and physiological signals (nutrient deficiency, temperature, stress etc.). Once activated, Spo0A-P controls the expression of many genes and operons at the onset of sporulation, including those involved in the switch to asymmetric division. Thereafter, a cascade of cell type-specific RNA polymerase sigma factors controls gene expression in the forespore and in the mother cell.

Thirteen (16%) out of 80 analysed genes involved in the sporulation process were differentially expressed in the *fliC* mutant compared to the wild-type strain (Table 3). All of them were up-regulated and correspond to late stages of the sporulation process (Stage V and VI). We found both genes encoding putative spore coat proteins (CDR0212-13) and genes encoding CotF, CotA and CotD (CDR0522, CDR1511 and CDR2291), recently demonstrated as spore coat proteins in *C. difficile* [52]. In addition, genes encoding *bclA1* (CDR0337), *bclA2* (CDR3090) and *bclA3* (CDR3193), possibly involved in the exosporium architecture, were about 3-fold up-regulated. We noted that the *bclA1* gene of the R20291 strain has a point mutation compared to the 630 strain [53] and may encode a non-functional protein.

**Table 3:** Sporulation genes highly differentially expressed *in vivo* in the *fliC* mutant compared to strain R20291.

Gene ID	Gene ortholog	Name	Description	Microarray fold change
CDR0212	CD630_02130		Putative spore coat protein	6.88
CDR0213	CD630_02140		Conserved hypothetical protein	8.22
CDR0337	CD196_0351		Fragment of putative exosporium glycoprotein	3.22
CDR0522	CD630_05970	*cotF*	Spore-coat protein CotF	2.58
CDR0714	CD630_07830		Putative stage IV sporulation protein	4.08
CDR1476	CD630_15790		Two-component sensor histidine kinase, sporulation associated *spo0A*	2.46
CDR1511	CD630_16130	*cotA*	Spore outer coat layer protein CotA	2.88
CDR2289	CD630_23990		Conserved hypothetical protein	2.93
CDR2291	CD630_24010	*cotD*	Spore-coat protein CotD; manganese catalase	4.18
CDR2802	CD630_29670	*spoVFB*	Dipicolinate synthase subunit B	3.40
CDR3090	CD630_32300	*bclA2*	Putative exosporium glycoprotein	3.32
CDR3193	CD630_33490	*bclA3*	Putative exosporium glycoprotein	2.82
CDR3406	CD630_35690		Sporulation_specific protease	2.98

In a recent study, Janoir et al. showed that the sporulation process in strain 630 is accelerated *in vivo* in the monoxenic mouse model [54]. They showed that more than 50% of spores compared to vegetative cells were produced early during the infection (before 24 hours), which was consistent with the early expression of genes from early and late stages of the sporulation process [54]. We recently observed the same phenomenon with the R20291 strain in the monoxenic mouse model (unpublished results). Therefore, the results of our study suggest that *in vivo* sporulation may take place earlier in the *fliC* mutant compared to the parental strain.

**Figure 2:**
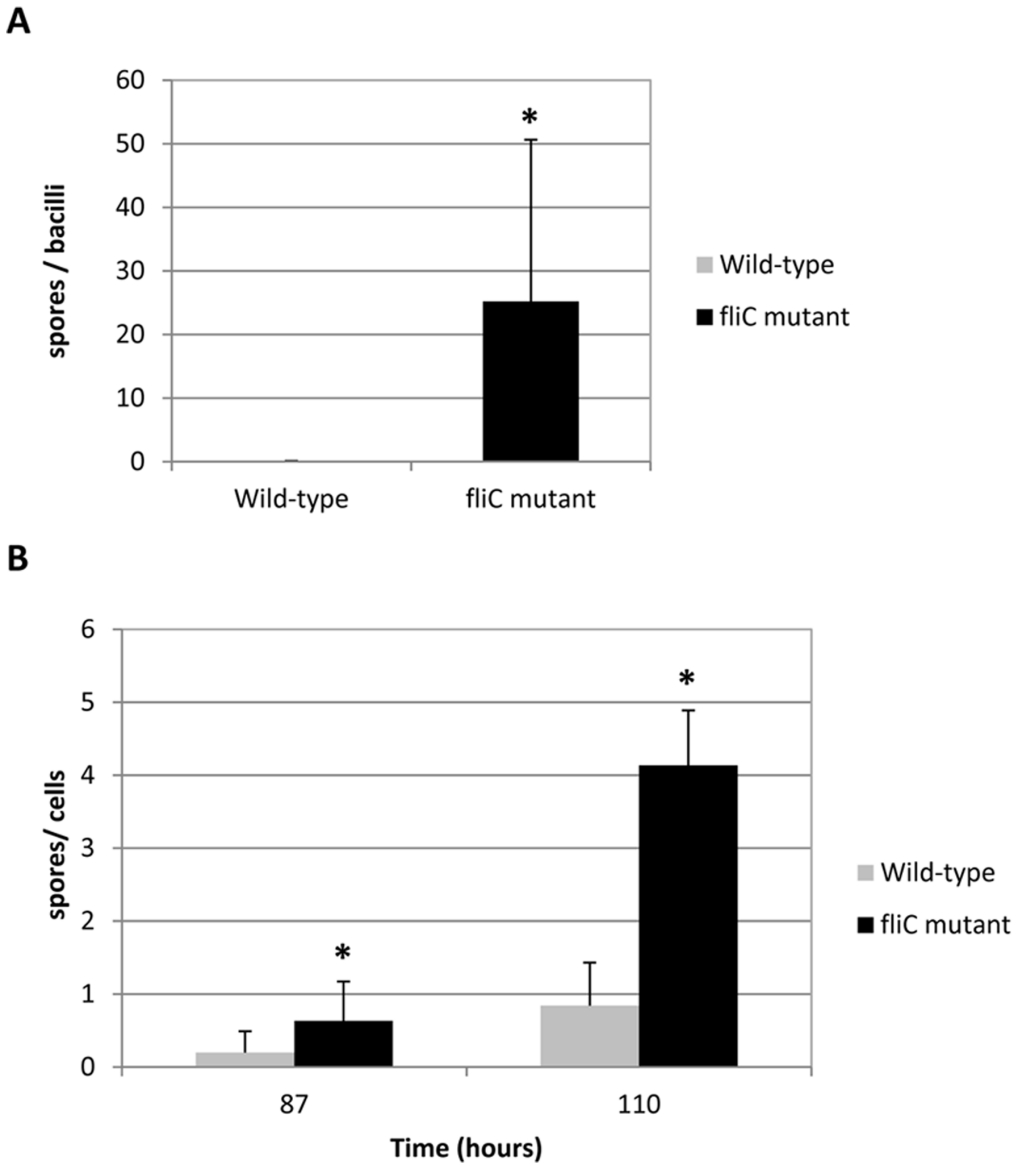
Ability of sporulation of the *fliC* mutant compared to wild-type R20291 *in vivo* and *in vitro*. (**A**) Groups of 6 axenic mice were infected by oral gavage route with 1×10^8^ *C. difficile* CFU. The *C. difficile* faecal vegetative cells and spores were measured by determining the concentration of CFU in faeces at 14 h post-infection by homogenising and plating on appropriated agar medium after heat shock (for spores) or not (Materials and Methods). Data represent the ratio of spores per vegetative cells. (**B**) Cultures in BHIS broth in anaerobic conditions were prepared from 2 successive subcultures as indicated in Materials and Methods. After heat shock, spores were quantified (CFU/ml) by performing serial dilutions and spread plating on BHIS agar supplemented with 0.1% bile salt taurocholate to induce germination. Data represent the ratio of spores per total cells. The presented values are the mean of 3 different cultures. Statistically significant difference is indicated by * for p<0,05.

In order to evaluate the impact of the *fliC* mutation on sporulation, we quantified the sporulation rates in faeces and caeca from mice at 14 h-post infection. In the faeces, the number of spores and the spore/bacilli ratio were significantly greater (p<0.05) for the *fliC* mutant than the wild-type strain (Figure 2A). In contrast, no difference in spore formation was observed in the caeca at 14 h post-infection (data not shown). We confirmed *in vitro* the impact of the absence of FliC on the sporulation rate. In fact, the spore/bacilli ration in BHIS was significantly greater (p<0.05) for the *fliC* mutant than the wild-type strain at 87 and 110 h after inoculation of culture (Figure 2B).

Taken together, these results suggest that inactivation of *fliC* may have induced a stress response culminating in the early production of spores. Thus, there is an inverse co-regulation between *C. difficile* flagella synthesis, motility and sporulation, as previously described for *B. subtilis* [55].

### Known and putative virulence factor genes

#### Toxins

increased toxicity was previously shown in the *fliC-*630Δ*erm* flagella mutant [35], [38]. Moreover, Baban et al. [38] showed by qRT-PCR that expression of the *tcdA* gene was strongly up-regulated (44-fold greater) *in vitro* in the *fliC-*630Δ*erm* mutant confirming that the regulation of the toxin in this *fliC*-mutant occurs at the level of the transcription. Surprisingly, our transcriptomic analysis revealed no differences in the toxin gene expression between the *fliC*-R20291 mutant and the wild-type strain, although qRT-PCR analysis showed that the transcription of the *tcdA* and *tcdB* genes, as well as the regulator gene *tcdC*, were approximately 2-fold lower in the *fliC* mutant compared to the wild-type strain (Table 4). In agreement with this result, no difference in cytotoxicity was observed *in vitro* when we compared the wild-type strain R20291 and its *fliC* mutant [38]. Thus, toxin gene regulation in absence of FliC is quite different between *C. difficile* strain 630 and strain R20291. Interestingly, these results suggest that the high mortality of mice infected by the *fliC* R20291 mutant could not be explained by an increase of toxin gene synthesis but by other genes whose expression is dependent on flagella.

**Table 4:** Known and putative virulence factor genes differentially expressed *in vivo i*n the *fliC* mutant compared to strain R20291.

Gene ID	Gene ortholog	Name	Description	Fold change (microarray)	Fold change (qRT-PCR)
CDR0584	CD630_06630	*tcdA*	Toxin TcdA	1	0.64
CDR0582	CD630_06600	*tcdB*	Toxin TcdB	1	0.59
CDR0585	CD630_06640	*tcdC*	Negative regulator of toxin gene expression	0.48	0.38
CDR0581	CD630_06590	*tcdR*	Alternative RNA polymerase sigma factors	1	ND
CDR0583	CD630_06610	*tcdE*	Holin-like pore-forming protein	1	ND
CDR2491	CD630_26040	*cdtA*	Fragment of ADP-ribosyltransferase CdtAB	1	2.49
CDR2492	CD630_26050	*cdtB*	Fragment of ADP-ribosyltransferase CdtAB	2.36	2.60
CDR2479	CD630_25920	*fbpA*	Fibronectin-binding protein A	1	0.03
CDR0195	CD630_01940	*groEL*	60 kDa chaperonin (Protein Cpn60) (GroEL protein)	1	0.36
CDR2676	CD630_27870	*cwp84*	Cell surface protein Cwp84	1	0.41
CDR0224	CD196_0237		Glucose-1-phosphate thymidylyltransferase	4.82	12.66
CDR2930	CD630_30910	*treA*	Trehalose-6-phosphate hydrolase	24.38	177.63
CDR0152	CD630_01530	*hpdB*	4-hydroxyphenylacetate decarboxylase, catalytic subunit (p-cresol production)	1	1.67
CDR0154	CD630_01550	*hpdA*	4-hydroxyphenylacetate decarboxylase, activating subunit (p-cresol production)	3.11	1.72
CDR0598	CD630_06750	*cfsT*	CsfT sigma factor (ECF sigma factors family)	5.02	3.37
CDR2092	CD196_2049		Lipoprotein	2.31	ND
CDR0440	CD196_0454		Hemagglutinin/adhesin	0.11	ND

ND: non determined.

We noted that the genes *cdtA* and *cdtB*, coding for binary toxin (CDT), were 2-fold up-regulated in the *fliC*-R20291 mutant compared to wild-type strain (Table 4). A tentative role for CDT in virulence has recently been reported [56]. However, any correlation between the observed up-regulation of binary toxin genes in the *fliC* mutant and increased virulence remains to be determined.

#### Membrane proteins

with the exception of *cwp66* (2.87-fold up-regulated), microarray results revealed that there was no significant differential expression for genes encoding known adhesins or cell wall proteins between the *fliC*-R20921 mutant and the parental strain. However, when we analysed the level of expression of these genes by qRT-PCR, a down-regulation of some genes was observed (Table 4). Among these we found *fbpA*, *groEL* and *cwp84*, which are known to be involved in *C. difficile* colonization [57]. Since variations of gene expression were not high enough to be detected by microarrays, qRT-PCR results indicate that *fliC* inactivation could be correlated to the down-regulation of several important *C. difficile* adhesins.

Based on our transcriptomic analyses, FliC and several other adhesins might be co-regulated in order to enhance the colonization process. Thus, the down-regulation of these adhesins in response to the *fliC* inactivation could explain the low colonization rate of the *fliC* R20291 mutant compared to the wild-type strain in the dixenic mice, as previously reported by Baban et al. [38]. Moreover, unlike the 630Δ*erm* strain, flagella mutants of R20291 are less adherent *in vitro* than the parental strain [38]. So, we can hypothesise that the absence of FliC in R20291 limits the contact of the bacteria to the epithelial surfaces leading to a down-regulation of some adhesion factors and toxins. Therefore, the hypervirulence of the *fliC* mutant must be due to expression of other as yet unknown factors in absence of FliC, which could play a role in induction of a deleterious host inflammatory response. Thus, an *in vivo* phase variation of the flagella in the R20291 strain might be co-regulated with other virulence factors during the course of the infection. Further work is needed to understand the involvement of the flagella regulon of R20291 in the expression of toxins, surface adhesins and other putative virulence factors during this process.

Finally, several surface-associated protein genes, such as lipoprotein (CDR2092) and hemagglutinin/adhesin (CDR0440) genes are up- and down-regulated, respectively in the *fliC*-R20921 mutant compared to the parental strain (Table 4). Lipoproteins have been shown to play key roles in adhesion to host cells, modulation of inflammatory processes, and translocation of virulence factors into host cells [58]. The hemagglutinin gene was identified as specific to PCR-ribotype 027 strains, such as CD196 and R20291, and the corresponding protein was specifically found in the secretome of these strains [36], [59]. This protein probably plays an important role in the pathogenesis of PCR-ribotype 027 strains. Co-regulation of both genes by FliC could play a role in the modulation of the pathogenesis of *C. difficile* during infection.

### Metabolism and adaptation genes

Bacterial metabolism is an important feature to ensure the survival and adaptation to the environment. Some genes, which could play a role in the adaptation process of *C. difficile* in the intestinal tract, were differentially expressed in the *fliC* mutant compared to the parental R20291 strain. These genes include three genes unique to PCR-ribotype 027 strains that reside just upstream of the flagella F1 region (CDR0224-26), which encode enzymes involved in the synthesis of L-rhamnose and are highly up-regulated (3-5-fold) in the *fliC* mutant (Table 4). Carbohydrates such as L-rhamnose can act as energy sources [60]–[62] and could be an important contributor to the survival and adaptation of *C. difficile* within the GI tract.

Trehalose has been reported to be present as a storage carbohydrate in many bacteria and could be partially responsible for protection against environmental stresses, such as desiccation, high osmolarity and heat, thus conferring high resistance properties to bacteria [63]. In the *fliC*-R20921 mutant, the trehalose-6-phosphate hydrolase encoding gene *treA* (CDR2930), which catalyses conversion of trehalose to glucose, was strongly up-regulated (24-fold, Table 4), suggesting a possible role for this carbohydrate in the increased virulence of the *fliC*-R20921 mutant.

The catabolism of tyrosine is also affected in the *fliC* R20291 mutant. In fact, the *hpdBCA* operon (CDR0152-54) was up-regulated in the *fliC* mutant compared to wild-type strain (Table 4). This operon encodes components of *p*-hydroxyphenylacetate decarboxylase, an enzyme complex which catalyses the decarboxylation of *p*-hydroxyphenylacetate, a tyrosine degradation product, to yield the bacteriostatic compound, *p*-cresol. The weak but significant up-regulation observed for the *hpdBCA* operon in the *fliC* mutant, could lead to an increased *in vivo* production of *p*-cresol. Because *p*-cresol is well tolerated by *C. difficile*, it has been suggested that it provides the bacterium with a competitive advantage over other intestinal microflora and consequently contributes to virulence [64]–[66]. In absence of micro-biota in monoxenic mice, increasing *p*-cresol synthesis by the *fliC* mutant could contribute to the toxicity and/or inflammation of the host intestinal epithelium.

### Regulatory genes

Many genes encoding transcriptional regulators are present in the genome of *C. difficile* (10% of the total CDSs), which include more than 40 two-component regulatory systems [67]. However, very few regulatory genes, including only 2 two-component systems (CDR0591-0592), were differentially expressed in the *fliC* mutant, although most of them are positively regulated. Among the transcriptional regulators significantly up-regulated (Tables S3 and S4 in File S1), we found the AgrACBD and Fur encoding genes (CDR3189-3187 and CDR1127, respectively) and regulators belonging to the GatR families (CDR1936 and CDR2929). The *agr* locus generally plays a key role in controlling gene expression in several Gram-positive pathogens. In *C. difficile*, it was recently demonstrated that the *agr* locus modulates known *C. difficile* virulence factors such as flagella and toxins, and as yet uncharacterized two-component regulatory systems [68]. In addition, the *agr* locus has a contributory role in colonization and relapse of epidemic *C. difficile* 027 *in vivo* [68]. Originally, Fur was identified as an iron-dependent repressor of genes involved in the acquisition of iron [69]. However, Fur can also positively regulate gene expression in both low- and high-iron conditions, among which we can find important virulence determinants [70]. The metabolite-responsive GntR family of regulators is widely distributed throughout prokaryotes where they regulate many diverse biological processes, such as motility, development of antibiotic resistance, plasmid transfer and virulence [71]. All these regulators affected by the inactivation of the *fliC* gene in the R20291 strain, in addition to others, like transcriptional regulators sigma 54-dependent or belonging to the CarD family, strengthen the idea that flagellin regulation is intimately involved in many different cellular processes, which might contribute to the exacerbated virulence of the *fliC*-R20921 mutant.

### Specific R20291 genes

PCR-ribotype 027 strain R20291 is known to possess a number of genes which are absent in the 630 strain [36], [53]. As a group, PCR-ribotype 027 strains possess 234 additional genes, 47 of which are only present in strain R20291. Among these unique genes, 43 of the 234 and 4 of the 47 genes, respectively, are differentially expressed in the *fliC*-R20921 mutant (Table S3 in File S1). These additional genes could explain the difference observed between the *fliC* mutants of the R20291 and 630 strains, which react differently in the gnotobiotic mice model [38].

Among the 43 PCR-ribotype 027-specific genes differentially expressed in *fliC* mutant, two encode proteins which are secreted [59]: CDR0440 encodes an hemagglutinin/adhesin (cited above) and CDR2278 encodes a putative peptidoglycan-binding/hydrolysing protein (Table S3 in File S1). Both proteins could be involved in enhanced virulence of the *fliC* mutant. Further studies are necessary to understand their functional roles.

Other unique genes of the R20291 strain which are differentially expressed in the *fliC* mutant include a transcriptional regulator, a toxin-antitoxin system belonging to the conjugative transposon Tn6104 and an unknown protein encoded by CDR1747, CDR1759–1760 and CDR1774, respectively (Table S3 in File S1). Whether these factors contribute to virulence remains to be determined.

### Common genes differentially expressed in both *in vitro* and *in vivo* transcriptomic analyses

When we compared the *in vivo* and *in vitro* transcriptomic analyses performed on the *fliC*-R20291 mutant and its parental strain, we observed only 36 common genes were differentially expressed (Table S4 in File S1). Among them, we found genes encoding glucose PTS-specific IIABC components (CDR2554–2555), the cobalt transport protein CbiM (CDR0329), the flagellar motor protein FliG and the flagellar assembly protein FliH (CDR0253 and CDR0252, respectively), the ferric uptake regulator Fur (CDR1127) and several unknown proteins (Table S4 in File S1). The low number of genes that exhibited a similar pattern of differential expression between the *in vitro* and *in vivo* experiments shows that the dissimilar environmental conditions encountered during *in vivo* and *in vitro* conditions trigger quite different responses and underlines the importance of the *in vivo* approach to studying gene functions during *C. difficile* infection.

## Conclusions

In order to understand the reasons for the increased virulence of the *fliC* R20291 mutant, recently reported by Baban et al. [38], we compared the global gene expression profiles of the *fliC* mutant and the parental strain. The differences observed in the *fliC* mutant were shown to be a consequence of the disruption of the *fliC* gene by the demonstration that the trans *fliC*-complemented strain had the same transcriptomic profile as the wild-type strain. Significantly, only 36 out of 310 genes differentially expressed in the *in vivo* grown cells were also differentially expressed in cells grown under laboratory conditions. This observation clearly demonstrates that the *in vivo* environment is quite distinct from the ‘laboratory flask’, leading to significantly different responses of the bacterial cell. We already knew that disruption of the *fliC* gene in *C. difficile* strain 630 induced a high mortality in hamsters and a modulation of toxin gene transcription, suggesting a co-regulation of genes encoding flagella and toxins. Although some differences were observed between the *fliC* mutant in 630 and R20291, here we provide additional data supporting the existence of a link between flagella synthesis and sporulation, adhesion, metabolism and to a lesser extent, toxin production.

In addition to their role in motility and chemotaxis, bacterial flagella are involved in infection through their roles in host-cell adhesion, cell invasion, auto-agglutination and formation of biofilms, as well as in the regulation and secretion of non-flagellar bacterial proteins involved in virulence. The reasons for the enhanced virulence of the *fliC*-R20291 mutant remain unclear. It is possible that in absence of flagella, bacteria do not establish the same kind of contact with their environment at the beginning of the infection and before reaching the infection site. This could expose the bacteria to stresses that they would not face if they were mobile, leading to the induction of other regulatory pathways responsible for hypervirulence. This could be the situation of the non-flagellated, but toxigenic, PCR-ribotype 078 epidemic *C. difficile* strains which still possess the late flagellar genes that might play a role in regulation on environment adaptation and virulence.

It is possible that the flagellin of *C. difficile* might participate in processes other than flagella formation and motility, as described for other bacterial pathogens. Here we provide experimental evidence that FliC from *C. difficile* R20291 could be involved in elaborate regulatory networks controlling virulence genes including genes of sporulation, toxin, surface-associated proteins and a large number of genes of unknown function that would intervene in the adaptation and survival processes of *C. difficile* in the intestinal tract. This is consistent with the recent work of El Meouche et al. [41] which provides an additional argument about the role of SigD, which controls flagellar synthesis, in regulating the expression of genes involved in motility, metabolism, regulation, and also of genes encoding TcdA and TcdB as well as TcdR, the positive regulator of the toxins. However, the mechanism by which this regulatory network controls synthesis of the flagella structure and the non-flagellar genes is still unknown.

Previous studies on *C. difficile* flagella have highlighted significant differences between different strains. For example, the UK outbreak strain R20291 synthetizes only a single flagellum whereas strain 630Δerm produces peritrichous flagella. Furthermore, flagellar mutants of R20291 lacking *fliC* and *fliD* genes adhere less strongly *in vitro* than the parental strain while these mutants in 630Δerm adhere more strongly than the respective parental strain [35], [39], [72]. Interestingly, in the case of R20291, toxicity seems unaffected in flagellar mutants whereas an increase in toxicity is observed for flagellar mutants of 630Δerm [72]. Therefore, presumably regulation processes between flagella and other genes are quite different in these strains and we would expect a quite different gene expression in flagellar mutants of R20291 compared to 630Δerm. Moreover, as previously reported, flagellar glycosylation might play an important role and could explain differences seen in the behaviour of different *C. difficile* strains. In the present study we demonstrate variations in gene expression after disruption of the *fliC* gene in R20291, but these variations remain to be analysed in other flagellated strains. Further analysis is necessary to decipher the differences in regulation processes between *C. difficile* strains.

Finally, we cannot rule out the hypothesis that a strong immune response of the host might explain the high mortality rate of the monoxenic mice observed with the *fliC* mutant. However, we did not explore this question and further investigations are needed to understand the impact of the host response in the high virulence of the *fliC* R20291 mutant, which will help our understanding of *C. difficile* pathogenesis.

Taken together, this work supports a co-regulation between flagellar and non-flagellar genes. Indeed, our comparative *in vitro* and *in vivo* transcriptomic analyses show that by disabling the structural component of the flagellum, the flagellin protein FliC, a large number of genes are differentially expressed, which could be related to the hypervirulence of *C. difficile* in the monoxenic mouse model. Thus, through the participation of FliC, flagellar and non-flagellar genes are differentially expressed during infection. This suggests that when bacteria have reached their ecological niche, the synthesis of flagella could be reduced or abolished, thus resulting in expression of other pathogenic factors and the exacerbation of virulence. This hypothesis is in agreement with the observed *in vivo* kinetics of gene expression in *C. difficile* strain 630 [38], which showed a progressive decrease in the expression of flagellar genes throughout infection. In conclusion, we provide the first genetic evidence of the role of flagellin FliC in the virulence of *C. difficile* strain R20291, which could be used to generate novel countermeasures against *C. difficile*-associated disease.

## Materials and Methods

### Bacterial strains and growth conditions

In this study we used the *C. difficile* R20291 wild-type strain and its isogenic *fliC* mutant obtained by ClosTron technology as described previously [38]. Both *C. difficile* strains were routinely cultured in Brain Heart Infusion (BHI) or peptone yeast (PY), agar or broth medium (Oxoid) under anaerobic conditions (10% CO_2_, 10% H_2_ and 80% N_2_.) at 37°C. *C. difficile* strains containing plasmid pMTL84151 were grown in BHI or PY medium supplemented with 15 µg ml^−1^ thiamphenicol. Cultures for sporulation assays were performed in BHIS (BHI containing 5 mg ml^−1^ yeast extract (AES) and 0.1% [w/v] L-cysteine (Merck) and germination was obtained in BHIS medium supplemented with 0.1% [w/v] bile salt taurocholate. For conjugation experiments, *E. coli* HB101 (RP4) strain was grown in Luria-Bertani (LB) medium supplemented with 100 µg ml^−1^ ampicillin and 25 µg ml^−1^ chloromphenicol. Transconjugants were grown on Columbia Cysteine agar supplemented with 0.4% *C. difficile* selective supplements (Oxoid) and 4% [v/v] defibrinated horse blood.

### Complementation of the *fliC* mutant

To complement the *fliC* mutant, a 973-bp fragment encompassing the *fliC* open reading frame and the *fliC* promoter region was amplified and cloned into the expression vector pMTL84151, as previously described [38]. The complementation plasmid was transferred into *C. difficile* via conjugation and transconjugants were verified by PCR.

### Motility assays

Cells from overnight cultures were stab-inoculated into soft agar (BHI with 0.4% agar) and incubated at 37°C in an anaerobe chamber. Forty eight hours later, motility was visually observed by diffuse growth spreading from the line of inoculation.

### Gnotobiotic mouse model

Animal care and animal experiments have been carried out in accordance with the Committee for Research and ethical Issues of the International Association for the study of pain (IASP). The animal experimentation protocol was approved by the Animal Welfare Committee of the Paris Sud University and animal experiments were performed according to the University Paris Sud guidelines for the husbandry of laboratory animals. C3H/HeN germ-free (6-8 weeks old), obtained from CDTA (CNRS Orléans, France), were housed in sterile isolators provided with sterilized bedding and received standard nutrients sterilized by irradiation, and water sterilized by autoclaving. Prior to infection with *C. difficile* strains, each animal was checked for germ-free status by Gram staining of faeces and by inoculating faeces in BHI and incubating broth for 48 h, either aerobically or anaerobically. Cultures of *C. difficile* for challenge were prepared for each mouse model as previously described [73]. Groups of 6 axenic mice were infected by oral gavage route with 1×10^8^ *C. difficile* CFUs of vegetative cells of *C. difficile* R20291 strain and *fliC* mutant obtained from a 9 h culture in PY media. The *C. difficile* faecal shedding (vegetative and spores) was measured by determining the concentration of *C. difficile* CFU in faeces during the course of infection by homogenising and plating faecal dilutions either on BHI (vegetative cells), or, after a heat shock treatment (60°C, 30 min) on BHI containing 0.1% of taurocholate sodium salt (spores).

### RNA extraction for *in vitro* conditions

Total RNA from the *in vitro* independent cultures was extracted from strains cultured in PY broth medium containing 15 µg ml^−1^ thiamphenicol. RNA was extracted at the early stationary phase (14 h growth) from four independent cultures of each strain by using the FastRNA Pro Blue Kit/FastPrep Instrument (QBiogene) according to the manufacturer's instructions. DNA contaminating the RNA samples was removed using a DNA freeTM DNase (Ambion). The RNA was further assessed by analysis on the Bionalyser Agilent 2100 and RNA 6000 Nano Reagents (Agilent) and by quantitative RT-PCR on housekeeping genes *gyrA* and *rpoA* (see Table S1 in File S1 for primer sequences).

### RNA extraction for *in vivo* conditions

Bacterial RNA was extracted from monoxenic mouse caeca. Six germ-free male C3H mice were challenged by oral route with 1×10^8^ CFUs of vegetative cells of *C. difficile* R20291 strain and *fliC* mutant obtained from a 9 h culture in PY media. The caeca of 14 h post-infection mice were excised, immersed in RNAProtect Bacteria Reagent (Qiagen) and sectioned longitudinally to obtain the caecal contents and mucosal scrapings. Fecal detritus was removed and after centrifugation, total bacterial RNA was isolated from caecal contents and colonic mucosa as above. Specific bacterial RNA was extracted using MICROBEnrich (Ambion) according to the manufacturer's instructions and RNA quality and quantity analyzed as described above. According to RNA quality and quantity, the four best samples per strain were chosen for transcriptomic analysis.

### Reverse transcription and microarray hybridizations

Oligonucleotide microarrays were designed using the OligoArray 2.0 software [74] and produced by Agilent. They contain 3,552 gene sequences in the form of oligonucleotides of 45 to 60 nucleotides in length. Each gene is mainly represented by two oligonucleotides. We were unable to design oligonucleotides for 41 genes. Probes were replicated twice on the array to reach a final density of 14,314 probes per array. Five hundred thirty-six positive controls and 894 negative controls were also included. The description of the microarray design was submitted to the NCBI Gene Expression Omnibus (GEO) database (http://www.ncbi.nlm.nih.gov/projects/geo/) with the accession number GPL15218. Purified bacterial mRNAs derived from *in vitro* or *in vivo* experiments were first treated with 50 U of RNase-free DNase I at 37°C for 30 min. Then, cDNA was produced from the RNA by RT-PCR and labeled using Superscript indirect cDNA labeling kit (Invitrogen). The microarray slides were hybridized competitively with each *in vivo* cDNA to allow further direct comparisons between all slides hybridized. A dye swap was performed for each hybridization. Arrays were scanned with a GenePix 4200 L dual-channel (635 nm and 532 nm) laser scanner (GenePix). All slides were then analyzed using the R and limma software. For each slide, background was corrected with the ‘normexp’ method [75] resulting in strictly positive values and reducing variability in the log ratios for genes with low levels of hybridization signal. Then, each slide was normalized with the ‘loess’ method [76]. To test for differential expression, the bayesian-adjusted t-statistics was used and the multiple testing corrections of Benjamini & Hochberg based on the false discovery rate (FDR) was performed [77]. A gene was considered differentially expressed when the P value was <0.05.

### Microarray data accession number

The complete experimental data sets from *in vivo* and *in vitro* analysis were deposited in the GEO database with the accession number GSE35727 (Reviewer access: https://www.ncbi.nlm.nih.gov/geo/query/acc.cgi?acc=GSE35727).

### Real-time reverse transcription PCR (qRT-PCR) analysis of gene expression

cDNA was prepared from 1 µg RNA using SuperScript III Reverse Transcriptase (Invitrogen) as described by the manufacturer. qRT-PCR was performed in triplicate in a 10 µl reaction volume containing 4 ng of cDNA, 5 µl of SSo Advanced SYBR Green Supermix (BIO-RAD) and 500 nM gene-specific primers. The cycling conditions comprised 30 sec polymerase activation at 95°C and 40 cycles at 95°C for 2 sec and 60°C for 5 sec. An additional step from a start at 65°C to 95°C (0.5°C/0.5 sec) was performed to establish a melting curve in order to verify the specificity of the real-time PCR reaction for each primer pair. Each assay included: a standard curve of eight serial dilution points of a mix of all cDNA samples (ranging from 10 ng to 4.6 pg), a no-template control, and 4 ng of each test cDNA. All PCR efficiencies were above 95%. The results were normalized using the geometric averaging of 4 reference genes (polIII, 16 S, rpoA and gyrA; see Table S1 in File S1 for primer sequences). Normalized relative quantities were calculated using the ΔΔCT method [78], [79]. Mann-Whitney test was performed using StatEL software to determine the significance difference (p<0.05).

### Sporulation assay

To ensure the lack of spores on inoculation of the sporulation medium, cultures in BHS broth in anaerobic conditions were prepared from 2 successive cultures by inoculating 1 in 100 at each time as previously described [80]. Briefly, overnight cultures in BHIS broth were used to inoculate a starter culture in BHIS broth 1∶100 dilution, which was grown to an OD_600_ of between 0.2 and 0.5. The sporulation medium was then inoculated 1∶100 with the starter culture. After heat shock treatment (60°C, 30 min), spores were quantified (CFU/ml) by performing serial dilutions and spread plating on BHIS agar supplemented with 0.1% bile salt taurocholate to induce germination. For *in vivo* sporulation assay, six germ-free male C3H mice were challenged by oral route with 1×10^8^ CFUs of vegetative cells of the *C. difficile* strains obtained from a 9 h culture in TY media (early stationary phase, spore rate less than 0.1% of vegetative cells). To measure spores, faeces were collected 14 h post-gavage and diluted in PBS in order to have 10 mg faeces ml^−1^. Appropriate dilutions were plated either on BHI to numerate vegetative cells, or, after a heat shock treatment (60°C, 30 min) on BHI containing 0.1% of taurocholate sodium salt to numerate the spores.

The caeca-adhering spores were also determined after sacrifice of mice 14 h post-gavage. Each caecum was washed eight times by gentle shaking in PBS buffer, weighed and placed in PBS to a final concentration of 10 mg caeca ml^−1^. The caecum was then homogenized with an Ultra-Turrax apparatus (IKA-Labortechnik) for 1 min at 13 500 r.p.m. The number of spores was then counted as described above. The data were represented as total spores per g faeces or per g caeca. Data were analysed by Mann-Whitney test. A statistically significant difference was considered to be p values of <0.05.

## Supporting Information

Figure S1 Kinetic of growth of the *fliC* mutant (FliC) compared to complemented FliC::*fliC* mutant and wild-type R20291 (027) *in vitro*.
(TIF)

File S1 Contains the files: Table S1 Primer pairs for qRT-PCR used in this study. Table S2 Genes analysed by qRT-PCR from *in vivo* experiments. Table S3 027- and R20291- specific genes highly differentially expressed *in vivo* in the *fliC* mutant compared to R20291 027 strain. Table S4 *In vivo*/*in vitro* common genes highly differentially expressed in the *fliC* mutant compared to R20291 027 strain.
(DOCX)

## References

[1] Cartman ST, Heap JT, Kuehne SA, Cockayne A, Minton NP (2010) The emergence of ‘hypervirulence’ in *Clostridium difficil*e. Int J Med Microbiol 300: 387–395. 20547099 10.1016/j.ijmm.2010.04.008

[2] Killgore G, Thompson A, Johnson S, Brazier J, Kuijper E, et al (2008) Comparison of seven techniques for typing international epidemic strains of *Clostridium difficile*: restriction endonuclease analysis, pulsed-field gel electrophoresis, PCR-ribotyping, multilocus sequence typing, multilocus variable-number tandem-repeat analysis, amplified fragment length polymorphism, and surface layer protein A gene sequence typing. J Clin Microbiol 46: 431–437. 18039796 10.1128/JCM.01484-07 PMC2238077

[3] Borgmann S, Kist M, Jakobiak T, Reil M, Scholz E, et al. (2008) Increased number of *Clostridium difficile* infections and prevalence of Clostridium difficile PCR ribotype 001 in southern Germany. Euro Surveill 13.. 19081002

[4] Brazier JS, Patel B, Pearson A (2007) Distribution of *Clostridium difficile* PCR ribotype 027 in British hospitals. Euro Surveill 12 : E070426 070422. 10.2807/esw.12.17.03182-en 17868609

[5] Indra A, Schmid D, Huhulescu S, Hell M, Gattringer R, et al (2008) Characterization of clinical *Clostridium difficile* isolates by PCR ribotyping and detection of toxin genes in Austria, 2006–2007. J Med Microbiol 57: 702–708. 18480326 10.1099/jmm.0.47476-0

[6] Kuijper EJ, Coignard B, Brazier JS, Suetens C, Drudy D, et al (2007) Update of *Clostridium difficile*-associated disease due to PCR ribotype 027 in Europe. Euro Surveill 12: E1–2. 10.2807/esm.12.06.00714-en 17991399

[7] Spigaglia P, Barbanti F, Dionisi AM, Mastrantonio P (2010) *Clostridium difficile* isolates resistant to fluoroquinolones in Italy: emergence of PCR ribotype 018. J Clin Microbiol 48: 2892–2896. 20554809 10.1128/JCM.02482-09 PMC2916588

[8] Goorhuis A, Bakker D, Corver J, Debast SB, Harmanus C, et al (2008) Emergence of *Clostridium difficile* infection due to a new hypervirulent strain, polymerase chain reaction ribotype 078. Clin Infect Dis 47: 1162–1170. 18808358 10.1086/592257

[9] Cheknis AK, Sambol SP, Davidson DM, Nagaro KJ, Mancini MC, et al (2009) Distribution of Clostridium difficile strains from a North American, European and Australian trial of treatment for *C. difficile* infections: 2005–2007. Anaerobe 15: 230–233. 19737618 10.1016/j.anaerobe.2009.09.001

[10] Genth H, Dreger SC, Huelsenbeck J, Just I (2008) *Clostridium difficile* toxins: more than mere inhibitors of Rho proteins. Int J Biochem Cell Biol 40: 592–597. 18289919 10.1016/j.biocel.2007.12.014

[11] Just I, Selzer J, von Eichel-Streiber C, Aktories K (1995) The low molecular mass GTP-binding protein Rho is affected by toxin A from *Clostridium difficile* . J Clin Invest 95: 1026–1031. 7883950 10.1172/JCI117747 PMC441436

[12] Just I, Selzer J, Wilm M, von Eichel-Streiber C, Mann M, et al (1995) Glucosylation of Rho proteins by *Clostridium difficile* toxin B. Nature 375: 500–503. 7777059 10.1038/375500a0

[13] Kuehne SA, Cartman ST, Heap JT, Kelly ML, Cockayne A, et al (2010) The role of toxin A and toxin B in *Clostridium difficile* infection. Nature 467: 711–713. 20844489 10.1038/nature09397

[14] Lyras D, O'Connor JR, Howarth PM, Sambol SP, Carter GP, et al (2009) Toxin B is essential for virulence of *Clostridium difficile* . Nature 458: 1176–1179. 19252482 10.1038/nature07822 PMC2679968

[15] Calabi E, Calabi F, Phillips AD, Fairweather NF (2002) Binding of *Clostridium difficile* surface layer proteins to gastrointestinal tissues. Infect Immun 70: 5770–5778. 12228307 10.1128/IAI.70.10.5770-5778.2002 PMC128314

[16] Hennequin C, Porcheray F, Waligora-Dupriet A, Collignon A, Barc M, et al (2001) GroEL (Hsp60) of *Clostridium difficil*e is involved in cell adherence. Microbiology 147: 87–96. 11160803 10.1099/00221287-147-1-87

[17] Waligora AJ, Hennequin C, Mullany P, Bourlioux P, Collignon A, et al (2001) Characterization of a cell surface protein of *Clostridium difficil*e with adhesive properties. Infect Immun 69: 2144–2153. 11254569 10.1128/IAI.69.4.2144-2153.2001 PMC98141

[18] Tasteyre A, Barc MC, Collignon A, Boureau H, Karjalainen T (2001) Role of FliC and FliD flagellar proteins of *Clostridium difficile* in adherence and gut colonization. Infect Immun 69: 7937–7940. 11705981 10.1128/IAI.69.12.7937-7940.2001 PMC98895

[19] Arora SK, Ritchings BW, Almira EC, Lory S, Ramphal R (1998) The *Pseudomonas aeruginosa* flagellar cap protein, FliD, is responsible for mucin adhesion. Infect Immun 66: 1000–1007. 9488388 10.1128/iai.66.3.1000-1007.1998 PMC108008

[20] Lillehoj EP, Kim BT, Kim KC (2002) Identification of *Pseudomonas aeruginosa* flagellin as an adhesin for Muc1 mucin. Am J Physiol Lung Cell Mol Physiol 282: L751–756. 11880301 10.1152/ajplung.00383.2001

[21] Eaton KA, Suerbaum S, Josenhans C, Krakowka S (1996) Colonization of gnotobiotic piglets by *Helicobacter pylori* deficient in two flagellin genes. Infect Immun 64: 2445–2448. 8698465 10.1128/iai.64.7.2445-2448.1996 PMC174096

[22] Rabaan AA, Gryllos I, Tomas JM, Shaw JG (2001) Motility and the polar flagellum are required for *Aeromonas caviae* adherence to HEp-2 cells. Infect Immun 69: 4257–4267. 11401962 10.1128/IAI.69.7.4257-4267.2001 PMC98495

[23] McSweegan E, Walker RI (1986) Identification and characterization of two *Campylobacter jejuni* adhesins for cellular and mucous substrates. Infect Immun 53: 141–148. 2873103 10.1128/iai.53.1.141-148.1986 PMC260088

[24] Dietrich C, Heuner K, Brand BC, Hacker J, Steinert M (2001) Flagellum of *Legionella pneumophila* positively affects the early phase of infection of eukaryotic host cells. Infect Immun 69: 2116–2122. 11254565 10.1128/IAI.69.4.2116-2122.2001 PMC98137

[25] Grant CC, Konkel ME, Cieplak W Jr, Tompkins LS (1993) Role of flagella in adherence, internalization, and translocation of *Campylobacter jejuni* in nonpolarized and polarized epithelial cell cultures. Infect Immun 61: 1764–1771. 8478066 10.1128/iai.61.5.1764-1771.1993 PMC280763

[26] Kirov SM, Castrisios M, Shaw JG (2004) Aeromonas flagella (polar and lateral) are enterocyte adhesins that contribute to biofilm formation on surfaces. Infect Immun 72: 1939–1945. 15039313 10.1128/IAI.72.4.1939-1945.2004 PMC375165

[27] Blair KM, Turner L, Winkelman JT, Berg HC, Kearns DB (2008) A molecular clutch disables flagella in the *Bacillus subtilis* biofilm. Science 320: 1636–1638. 18566286 10.1126/science.1157877

[28] Ethapa T, Leuzzi R, Ng YK, Baban ST, Adamo R, et al (2013) Multiple Factors Modulate Biofilm Formation by the Anaerobic Pathogen *Clostridium difficile* . J Bacteriol 195: 545–555. 23175653 10.1128/JB.01980-12 PMC3554014

[29] Konkel ME, Klena JD, Rivera-Amill V, Monteville MR, Biswas D, et al (2004) Secretion of virulence proteins from *Campylobacter jejuni* is dependent on a functional flagellar export apparatus. J Bacteriol 186: 3296–3303. 15150214 10.1128/JB.186.11.3296-3303.2004 PMC415756

[30] Poly F, Ewing C, Goon S, Hickey TE, Rockabrand D, et al (2007) Heterogeneity of a Campylobacter jejuni protein that is secreted through the flagellar filament. Infect Immun 75: 3859–3867. 17517862 10.1128/IAI.00159-07 PMC1951984

[31] Song YC, Jin S, Louie H, Ng D, Lau R, et al (2004) FlaC, a protein of *Campylobacter jejuni* TGH9011 (ATCC43431) secreted through the flagellar apparatus, binds epithelial cells and influences cell invasion. Mol Microbiol 53: 541–553. 15228533 10.1111/j.1365-2958.2004.04175.x

[32] Anderson JK, Smith TG, Hoover TR (2010) Sense and sensibility: flagellum-mediated gene regulation. Trends Microbiol 18: 30–37. 19942438 10.1016/j.tim.2009.11.001 PMC2818477

[33] Tasteyre A, Karjalainen T, Avesani V, Delmee M, Collignon A, et al (2001) Molecular characterization of fliD gene encoding flagellar cap and its expression among *Clostridium difficile* isolates from different serogroups. J Clin Microbiol 39: 1178–1183. 11230454 10.1128/JCM.39.3.1178-1183.2001 PMC87900

[34] Heap JT, Kuehne SA, Ehsaan M, Cartman ST, Cooksley CM, et al (2010) The ClosTron: Mutagenesis in Clostridium refined and streamlined. J Microbiol Methods 80: 49–55. 19891996 10.1016/j.mimet.2009.10.018

[35] Dingle TC, Mulvey GL, Armstrong GD (2011) Mutagenic analysis of the *Clostridium difficile* flagellar proteins, FliC and FliD, and their contribution to virulence in hamsters. Infect Immun 79: 4061–4067. 21788384 10.1128/IAI.05305-11 PMC3187235

[36] Stabler RA, He M, Dawson L, Martin M, Valiente E, et al (2009) Comparative genome and phenotypic analysis of *Clostridium difficile* 027 strains provides insight into the evolution of a hypervirulent bacterium. Genome Biol 10: R102. 19781061 10.1186/gb-2009-10-9-r102 PMC2768977

[37] Tasteyre A, Karjalainen T, Avesani V, Delmee M, Collignon A, et al (2000) Phenotypic and genotypic diversity of the flagellin gene (fliC) among *Clostridium difficile* isolates from different serogroups. J Clin Microbiol 38: 3179–3186. 10970353 10.1128/jcm.38.9.3179-3186.2000 PMC87348

[38] Baban ST, Kuehne SA, Barketi-Klai A, Cartman ST, Kelly ML, et al (2013) The Role of Flagella in *Clostridium difficile* Pathogenesis: Comparison between a Non-Epidemic and an Epidemic Strain. PLoS One 8: e73026. 24086268 10.1371/journal.pone.0073026 PMC3781105

[39] Aubry A, Hussack G, Chen W, KuoLee R, Twine SM, et al (2012) Modulation of toxin production by the flagellar regulon in *Clostridium difficile* . Infect Immun 80: 3521–3532. 22851750 10.1128/IAI.00224-12 PMC3457548

[40] Twine SM, Reid CW, Aubry A, McMullin DR, Fulton KM, et al (2009) Motility and flagellar glycosylation in *Clostridium difficile* . J Bacteriol 191: 7050–7062. 19749038 10.1128/JB.00861-09 PMC2772495

[41] El Meouche I, Peltier J, Monot M, Soutourina O, Pestel-Caron M, et al (2013) Characterization of the SigD regulon of C. difficile and its positive control of toxin production through the regulation of tcdR. PLoS One 8: e83748. 24358307 10.1371/journal.pone.0083748 PMC3865298

[42] Chevance FF, Hughes KT (2008) Coordinating assembly of a bacterial macromolecular machine. Nat Rev Microbiol 6: 455–465. 18483484 10.1038/nrmicro1887 PMC5963726

[43] Detmers FJ, Lanfermeijer FC, Poolman B (2001) Peptides and ATP binding cassette peptide transporters. Res Microbiol 152: 245–258. 11421272 10.1016/s0923-2508(01)01196-2

[44] Lazazzera BA (2001) The intracellular function of extracellular signaling peptides. Peptides 22: 1519–1527. 11587781 10.1016/s0196-9781(01)00488-0

[45] Hu KY, Saier MH Jr (2002) Phylogeny of phosphoryl transfer proteins of the phosphoenolpyruvate-dependent sugar-transporting phosphotransferase system. Res Microbiol 153: 405–415. 12405346 10.1016/s0923-2508(02)01339-6

[46] Poncet S, Milohanic E, Maze A, Nait Abdallah J, Ake F, et al (2009) Correlations between carbon metabolism and virulence in bacteria. Contrib Microbiol 16: 88–102. 19494580 10.1159/000219374

[47] Dupuy B, Sonenshein AL (1998) Regulated transcription of Clostridium difficile toxin genes. Mol Microbiol 27: 107–120. 9466260 10.1046/j.1365-2958.1998.00663.x

[48] Antunes A, Martin-Verstraete I, Dupuy B (2011) CcpA-mediated repression of *Clostridium difficile* toxin gene expression. Mol Microbiol 79: 882–899. 21299645 10.1111/j.1365-2958.2010.07495.x

[49] Antunes A, Camiade E, Monot M, Courtois E, Barbut F, et al (2012) Global transcriptional control by glucose and carbon regulator CcpA in Clostridium difficile. Nucleic Acids Res 40: 10701–10718. 22989714 10.1093/nar/gks864 PMC3510511

[50] Konings WN (2006) Microbial transport: adaptations to natural environments. Antonie Van Leeuwenhoek 90: 325–342. 17043914 10.1007/s10482-006-9089-3

[51] Stephenson K, Lewis RJ (2005) Molecular insights into the initiation of sporulation in Gram-positive bacteria: new technologies for an old phenomenon. FEMS Microbiol Rev 29: 281–301. 15808745 10.1016/j.femsre.2004.10.003

[52] Permpoonpattana P, Tolls EH, Nadem R, Tan S, Brisson A, et al (2011) Surface layers of *Clostridium difficile* endospores. J Bacteriol 193: 6461–6470. 21949071 10.1128/JB.05182-11 PMC3232898

[53] Stabler RA, Valiente E, Dawson LF, He M, Parkhill J, et al (2010) In-depth genetic analysis of Clostridium difficile PCR-ribotype 027 strains reveals high genome fluidity including point mutations and inversions. Gut Microbes 1: 269–276. 21327033 10.4161/gmic.1.4.11870 PMC3023608

[54] Janoir C, Deneve C, Bouttier S, Barbut F, Hoys S, et al (2013) Adaptive strategies and pathogenesis of *Clostridium difficile* from in vivo transcriptomics. Infect Immun 81: 3757–3769. 23897605 10.1128/IAI.00515-13 PMC3811758

[55] Molle V, Fujita M, Jensen ST, Eichenberger P, Gonzalez-Pastor JE, et al (2003) The Spo0A regulon of Bacillus subtilis. Mol Microbiol 50: 1683–1701. 14651647 10.1046/j.1365-2958.2003.03818.x

[56] Kuehne SA, Collery MM, Kelly ML, Cartman ST, Cockayne A, et al (2014) Importance of toxin A, toxin B, and CDT in virulence of an epidemic *Clostridium difficile* strain. J Infect Dis 209: 83–86. 23935202 10.1093/infdis/jit426 PMC3864386

[57] Deneve C, Janoir C, Poilane I, Fantinato C, Collignon A (2009) New trends in *Clostridium difficile* virulence and pathogenesis. Int J Antimicrob Agents 33 Suppl 1S24–28. 19303565 10.1016/S0924-8579(09)70012-3

[58] Kovacs-Simon A, Titball RW, Michell SL (2011) Lipoproteins of bacterial pathogens. Infect Immun 79: 548–561. 20974828 10.1128/IAI.00682-10 PMC3028857

[59] Boetzkes A, Felkel KW, Zeiser J, Jochim N, Just I, et al (2012) Secretome analysis of *Clostridium difficile* strains. Arch Microbiol 194: 675–687. 22398929 10.1007/s00203-012-0802-5

[60] Dong C, Major LL, Srikannathasan V, Errey JC, Giraud MF, et al (2007) RmlC, a C3′ and C5′ carbohydrate epimerase, appears to operate via an intermediate with an unusual twist boat conformation. J Mol Biol 365: 146–159. 17046787 10.1016/j.jmb.2006.09.063 PMC1805628

[61] Tsukioka Y, Yamashita Y, Oho T, Nakano Y, Koga T (1997) Biological function of the dTDP-rhamnose synthesis pathway in *Streptococcus mutans* . J Bacteriol 179: 1126–1134. 9023194 10.1128/jb.179.4.1126-1134.1997 PMC178808

[62] Yamashita Y, Tomihisa K, Nakano Y, Shimazaki Y, Oho T, et al (1999) Recombination between gtfB and gtfC is required for survival of a dTDP-rhamnose synthesis-deficient mutant of *Streptococcus mutans* in the presence of sucrose. Infect Immun 67: 3693–3697. 10377163 10.1128/iai.67.7.3693-3697.1999 PMC116568

[63] Strom AR, Kaasen I (1993) Trehalose metabolism in *Escherichia coli*: stress protection and stress regulation of gene expression. Mol Microbiol 8: 205–210. 8391102 10.1111/j.1365-2958.1993.tb01564.x

[64] Dawson LF, Donahue EH, Cartman ST, Barton RH, Bundy J, et al (2011) The analysis of para-cresol production and tolerance in *Clostridium difficile* 027 and 012 strains. BMC Microbiol 11: 86. 21527013 10.1186/1471-2180-11-86 PMC3102038

[65] Dawson LF, Stabler RA, Wren BW (2008) Assessing the role of p-cresol tolerance in *Clostridium difficile* . J Med Microbiol 57: 745–749. 18480332 10.1099/jmm.0.47744-0

[66] Selmer T, Andrei PI (2001) p-Hydroxyphenylacetate decarboxylase from *Clostridium difficile*. A novel glycyl radical enzyme catalysing the formation of p-cresol. Eur J Biochem 268: 1363–1372. 11231288 10.1046/j.1432-1327.2001.02001.x

[67] Sebaihia M, Wren BW, Mullany P, Fairweather NF, Minton N, et al (2006) The multidrug-resistant human pathogen *Clostridium difficile* has a highly mobile, mosaic genome. Nat Genet 38: 779–786. 16804543 10.1038/ng1830

[68] Martin MJ, Clare S, Goulding D, Faulds-Pain A, Barquist L, et al (2013) The agr locus regulates virulence and colonization genes in *Clostridium difficile* 027. J Bacteriol 195: 3672–3681. 23772065 10.1128/JB.00473-13 PMC3754575

[69] Litwin CM, Calderwood SB (1993) Role of iron in regulation of virulence genes. Clin Microbiol Rev 6: 137–149. 8472246 10.1128/cmr.6.2.137 PMC358274

[70] Johnson M, Sengupta M, Purves J, Tarrant E, Williams PH, et al (2011) Fur is required for the activation of virulence gene expression through the induction of the sae regulatory system in *Staphylococcus aureus* . Int J Med Microbiol 301: 44–52. 20705504 10.1016/j.ijmm.2010.05.003 PMC2994983

[71] Hoskisson PA, Rigali S (2009) Chapter 1: Variation in form and function the helix-turn-helix regulators of the GntR superfamily. Adv Appl Microbiol 69: 1–22. 19729089 10.1016/S0065-2164(09)69001-8

[72] Baban ST, Kuehne SA, Barketi-Klai A, Hardie KR, Kansau I, et al. (2013) The role of flagella in *Clostridium difficile* pathogenesis: Comparison of a non-epidemic and an epidemic strain. PLoS One (Submited). 10.1371/journal.pone.0073026 PMC3781105 24086268

[73] Barketi-Klai A, Hoys S, Lambert-Bordes S, Collignon A, Kansau I (2011) Role of fibronectin binding protein A in *Clostridium difficile* intestinal colonization. J Med Microbiol. 10.1099/jmm.0.029553-0 21349990

[74] Rouillard JM, Zuker M, Gulari E (2003) OligoArray 2.0: design of oligonucleotide probes for DNA microarrays using a thermodynamic approach. Nucleic Acids Res 31: 3057–3062. 12799432 10.1093/nar/gkg426 PMC162330

[75] Breitling R, Amtmann A, Herzyk P (2004) Iterative Group Analysis (iGA): a simple tool to enhance sensitivity and facilitate interpretation of microarray experiments. BMC Bioinformatics 5: 34. 15050037 10.1186/1471-2105-5-34 PMC403636

[76] Smyth GK, Speed T (2003) Normalization of cDNA microarray data. Methods 31: 265–273. 14597310 10.1016/s1046-2023(03)00155-5

[77] Benjamini Y, Hochberg Y (1995) Controlling the false discovery rate: a practical and powerful approach to multiple testing. J R Stat Soc Ser B 57: 289–300.

[78] Hellemans J, Mortier G, De Paepe A, Speleman F, Vandesompele J (2007) qBase relative quantification framework and software for management and automated analysis of real-time quantitative PCR data. Genome Biol 8: R19. 17291332 10.1186/gb-2007-8-2-r19 PMC1852402

[79] Livak KJ, Schmittgen TD (2001) Analysis of relative gene expression data using real-time quantitative PCR and the 2(-Delta Delta C(T)) Method. Methods 25: 402–408. 11846609 10.1006/meth.2001.1262

[80] Burns DA, Heeg D, Cartman ST, Minton NP (2011) Reconsidering the sporulation characteristics of hypervirulent *Clostridium difficile* BI/NAP1/027. PLoS One 6: e24894. 21949780 10.1371/journal.pone.0024894 PMC3174218

