# Supplementary figures and images for: The Flagellin FliC of Clostridium difficile Is Responsible for Pleiotropic Gene Regulation during In Vivo Infection
Source: PLoS One. 2014 May 19;9(5):e96876. doi: 10.1371/journal.pone.0096876 (PMC4026244; doi:10.1371/journal.pone.0096876)

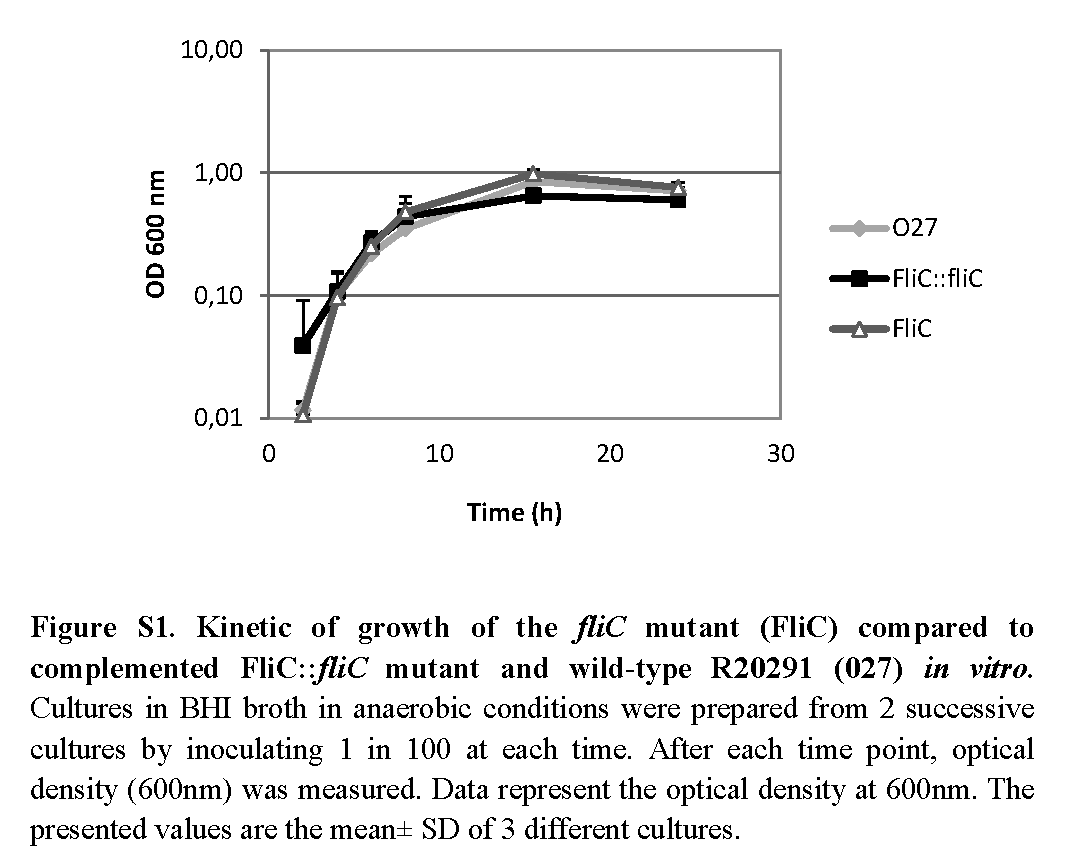

Supplement: Figure S1 — Kinetic of growth of the fliC mutant (FliC) compared to complemented FliC::fliC mutant and wild-type R20291 (027) in vitro. (TIF) [file pone.0096876.s001.tif]
