# Supplementary material for: The Flagellin FliC of Clostridium difficile Is Responsible for Pleiotropic Gene Regulation during In Vivo Infection
Source: PLoS One. 2014 May 19;9(5):e96876. doi: 10.1371/journal.pone.0096876 (PMC4026244; doi:10.1371/journal.pone.0096876)
Supplement: File S1 — Contains the files: Table S1 Primer pairs for qRT-PCR used in this study. Table S2 Genes analysed by qRT-PCR from in vivo experiments. Table S3 027- and R20291- specific genes highly differentially expressed in vivo in the fliC mutant compared to R20291 027 strain. Table S4 In vivo/in vitro common genes highly differentially expressed in the fliC mutant compared to R20291 027 strain. (DOCX) [file pone.0096876.s002.docx]

Supplementary data to:

**The flagellin FliC of *Clostridium difficile* is responsible for pleiotropic gene regulation during in vivo infection.**

**Table S1.** Primer pairs for qRT-PCR used in this study

| **Gene ID** | **Primers** | **Sequences (5’-3’)** | **Amplicon size (bp)** |
| --- | --- | --- | --- |
| CDR0238 | fliD-F | AACTGGTTCGTCATCAGACATT | 127 |
|  | fliD-R | TACACTTGCCACTTTGTTTCCA |  |
| CDR3187 | agr2-F | TGGACATACAAATGAAAACAATAAA | 147 |
|  | agr2-R | ACCTCATAACCTTCTTGCATAAACT |  |
| CDR0598 | csfT-F | TATTGGTTGGTGTGACGTTTT | 101 |
|  | csfT-R | AAAGACTACTTCCGATACCTTTTTC |  |
| CDR2930 | treA-F | GGTTTTCGTTTAGATGTAGTCAACC | 105 |
|  | treA-R | ACCATCAGCGTAATGAGTTCTTC |  |
| CDR0224 | rham-F | TATTTTAGCAGGAGGGTCTGGT | 118 |
|  | rham-R | CAACATAAGAACCGACATAGGGTAA |  |
| CDR0783 | oppB-F | TGCAAATTAGGAACAATAACCAACT | 133 |
|  | oppB-R | ATATACTGGACTCTTCTCCCATTGA |  |
| CDR2927 | pts1-F | AAAATGATGATGTATGTTTTGGTTT | 143 |
|  | pts1-R | TGTCAGTAGTTGCGTTCTTCTTATT |  |
| CDR2862 | pts2-F | TAGAATTAGAAAATGGGATGGAACT | 92 |
|  | pts2-R | CATACTCACCAATGCCTCAAAA |  |
| CDR3138 | pts3-F | TGGGAATTGCAGGGATAGG | 145 |
|  | pts3-R | AGAGATTGTGCTAATACTGCTACTGG |  |
| CDR0585 | tcdC-F | ACCATGAGGAGGTCATTTCTAATC | 128 |
|  | tcdC-R | AGCTTTCTTTTCGTCGTCTTTC |  |
| CDR0154 | hpdA-F | TGCAAATCCAGAAAGTTGGAC | 108 |
|  | hpdA-R | GCACCATTTTTACACTTACCATGAC |  |
| CDR0152 | hpdB-F | CGCCATTATAGAGGTTCTTTTCC | 150 |
|  | hpdB-R | AGTAACATTTCCTCCACCAGACC |  |
| CDR1579 | HK-F | TGGAACAGCAAGGGATATAAGTAG | 101 |
|  | HK-R | GCGTTCTAAGTTCATGGGATAA |  |
| CDR1105 | CDR1105-F | TTGATTGTTTATGGGTTTTTGATAGA | 147 |
|  | CDR1105-R | CCTTATCTGTGCTACATAGTTGTTTTG |  |
| CDR0212 | coat-F | GCATCTGCCAATATGTATCTTAATTCT | 118 |
|  | coat-R | TTAATTCTGTAAGTGCTGTGTGACC |  |
| CDR2491 | cdtA-F | GTCCAGTAAATAATCCTAACCCAGA | 127 |
|  | cdtA-R | AAACCAAATTCTTGAGGACCAG |  |
| CDR2492 | cdtB-F | ACCCAAAGTTGATGTCTGATTG | 132 |
|  | cdtB-R | CTGCAAAACTATCTTCCCACTT |  |
| CDR0584 | tcdA-F | GTCGGATTGCAAGTAATTGACAATA | 140 |
|  | tcdA-R | TAACAGTCTGCCAACCTTTTGAGA |  |
| CDR2479 | fbpA-F | AGTTCGTCAAGTTTTACCTGGTC | 120 |
|  | fbpA-R | GGTCCTTCCAATTCCTCTAGGT |  |
| CDR0195 | groEL-F | GAAAAGGAATACAAAAAGCAGTAACA | 114 |
|  | groEL-R | CATCACCAGCAGATATAGAAGCAA |  |
| CDR2676 | cwp84-F | TGG GCA ACT GGT GGA AAA TA | 151 |
|  | cwp84-R | TAG TTG CAC CTT GTG CCT CA |  |
| CDR2864 | malY-F | AGGATTTGCTTCCTTTTACAATTTC | 96 |
|  | malY-R | CCAAAACTCTATGATTTAACCTTTCC |  |
| CDR0785 | oppA-F | GAAAAATGGGACAAAGGTCA | 111 |
|  | oppA-R | AGCCTTATCATCTGGAACAATC |  |
| CDR0582 | tcdB-F | CACTTCTTTTCAGCACCATCA | 160 |
|  | tcdB-R | CTGGTGTCCATCCTGTTTCC |  |
| *dna polIII* | polIII-F | CCCAACTCTTCGCTAAGCAC | 149 |
|  | polIII-R | TCCATCTATTGCAGGGTGGT |  |
| *rrs* (*16S*) | rrs-F | GTGCCTCAGCGTCAGTTACA | 119 |
|  | rrs-R | GGGAGACTTGAGTGCAGGAG |  |
| *rpoA* | rpoA-F | TTTGACCAACTCTTGTGTTTTCC | 133 |
|  | rpoA-R | TAAAGGTAGAGGTTATGTTTCTGCT |  |
| *gyrA* | gyrA-F | TCCTTTACCAGCTCTTATTTGACTT | 120 |
|  | gyrA-R | TTTTGTTGTGTCTATGAACCTTTGT |  |
| *gluD* | gluD-F | TTCCACCTTTACCTCCACCA | 134 |
|  | gluD-R | ATGCAGTAGGGCCAACAAAA |  |
| *rpsJ* | rpsJ-F | GTCTTAGGTGTTGGATTAGCT | 150 |
|  | rpsJ-R | GATCACAAGTTTCAGGACCTG |  |

**Table S2.** Genes analysed by qRT-PCR from *in vivo* experiments.

| **Gene ID** | **Name** | **Fold change (microarray)** | **Fold change (qRT-PCR)** |
| --- | --- | --- | --- |
| CDR0238 | *fliD* | 5.05 | 9.85 |
| CDR2479 | *fbpA* | 1 | 0.03 |
| CDR0195 | *groEL* | 1 | 0.36 |
| CDR2676 | *cwp84* | 1 | 0.41 |
| CDR2927 | *pts1* | 51.25 | 70.15 |
| CDR2862 | *pts2* | 0.07 | 0.04 |
| CDR2864 | *malY* | 0.05 | 0.01 |
| CDR3138 | *pts3* | 11.76 | 17.63 |
| CDR0785 | *oppA* | 0.06 | 0.03 |
| CDR0783 | *oppB* | 0.07 | 0.06 |
| CDR0224 | *rham* | 4.82 | 12.66 |
| CDR2930 | *treA* | 24.38 | 177.63 |
| CDR0152 | *hpdA* | 3.11 | 1.72 |
| CDR0154 | *hpdB* | 1 | 1.67 |
| CDR3187 | *agr2* | 4.89 | 4.26 |
| CDR0584 | *tcdA* | 1 | 0.64 |
| CDR0582 | *tcdB* | 1 | 0.59 |
| CDR0585 | *tcdC* | 0.48 | 0.38 |
| CDR1105 | *-* | 3.49 | 5.65 |
| CDR0212 | *(coat)* | 6.88 | 6.66 |
| CDR1579 | *(HK)* | 2.46 | 2.67 |
| CDR0598 | *csfT* | 5.02 | 3.37 |
| CDR2491 | *cdtA* | 1 | 2.49 |
| CDR2492 | *cdtB* | 2.36 | 2.6 |

**Table S3.** 027- and R20291- specific genes highly differentially expressed *in vivo* in the *fliC* mutant compared to R20291 027 strain.

| **Gene ID** | **Gene orthologous** | **Description** | **Microarray fold change** |
| --- | --- | --- | --- |
| CDR0045 | CD196_0057 | Hypothetical protein | 2.77 |
| CDR0224 | CD196_0237 | Glucose-1-phosphate thymidylyltransferase | 4.82 |
| CDR0225 | CD196_0238 | dTDP-4-dehydrorhamnose 3,5-epimerase | 3.09 |
| CDR0226 | CD196_0239 | dTDP-glucose 4,6-dehydratase | 5.91 |
| CDR0243 | CD196_0256 | Glycosyl transferase group 2 family protein | 2.19 |
| CDR0244 | CD196_0257 | Hypothetical protein | 2.77 |
| CDR0440 | CD196_0454 | Cell surface protein ( hemagglutinin/adhesin) | 0.11 |
| CDR0551 | CD196_0569 | ABC transporter, ATP-binding protein/permease | 3.73 |
| CDR1429 | CD196_1454 | Hypothetical protein | 2.97 |
| CDR1430 | CD196_1455 | Hypothetical protein | 3.08 |
| CDR1439 | CD196_1464 | Hypothetical protein | 0.33 |
| CDR1443 | CD196_1468 | Hypothetical protein | 2.60 |
| CDR1447 | CD196_1472 | Hypothetical protein | 3.67 |
| CDR1448 | CD196_1473 | Hypothetical protein | 2.71 |
| CDR1462 | CD196_1487 | Hypothetical protein | 3.26 |
| CDR2092 | CD196_2049 | Lipoprotein | 2.31 |
| CDR2275 | CD196_2228A | Putative beta-lactamase inducer | 2.38 |
| CDR2278 | CD196_2230 | Peptidoglycan-binding/hydrolysing protein | 3.62 |
| CDR2760 | CD196_2713 | Two-component system, sensor histidine kinase | 2.02 |
| CDR2908 | CD196_2861 | Hypothetical protein | 2.75 |
| CDR2909 | CD196_2862 | Type I restriction enzyme R subunit | 2.51 |
| CDR2964 | CD196_2917 | Transcriptional regulator, PadR-like family | 2.34 |
| CDR2981 | CD196_2934 | Spermidine/putrescine ABC transporter ATP-binding subunit | 3.39 |
| CDR2982 | CD196_2935 | Hypothetical protein | 1.94 |
| CDR2983 | CD196_2936 | Putative ABC-type transport system, periplasmic component-like protein precursor | 2.80 |
| CDR2990 | CD196_2943 | Hypothetical protein | 2.89 |
| CDR2994 | CD196_2947 | CRISPR-associated helicase cas3 | 1.93 |
| CDR2995 | CD196_2948 | CRISPR-associated autoregulator | 2.12 |
| CDR2996 | CD196_2949 | Hypothetical protein | 2.24 |
| CDR2998 | CD196_2951 | CRISPR-associated protein cas5 family | 2.45 |
| CDR3185 | CD196_3139 | ABC transporter, ATP-binding protein | 2.1 |
| CDR3187 | CD196_3141 | Accessory gene regulator | 4.89 |
| CDR3188 | CD196_3142 | Sensor histidine kinase virs | 3.13 |
| CDR3189 | CD196_3143 | LytR family DNA-binding response regulator | 3.3 |
| CDR3278 | CD196_3232 | Putative exported protein precursor | 4.25 |
| CDR3281 | CD196_3235 | Transposon Tn21 resolvase | 3.65 |
| CDR3285 | CD196_3239 | Hypothetical protein | 5.45 |
| CDR3286 | CD196_3240 | Hypothetical protein | 3.09 |
| CDR3455 | CD196_3409 | Hypothetical protein | 2.91 |
| CDR3456 | CD196_3410 | Hypothetical protein | 2.95 |
| CDR3457 | CD196_3411 | Hypothetical protein | 3.39 |
| CDR3461 | CD196_3415 | Chloramphenicol O-acetyltransferase | 3.39 |
| CDR3469 | CD196_3423 | Transcriptional regulator, TetR family | 3.38 |
| CDR1747 |  | Putative transcriptional regulator | 2.02 |
| CDR1759 |  | Putative toxin-antitoxin system | 3.40 |
| CDR1760 |  | Putative toxin-antitoxin system | 2.90 |
| CDR1774 |  | Hypothetical protein | 2.97 |

**Table S4.** *In vivo* / *in vitro* common genes highly differentially expressed in the *fliC* mutant compared to R20291 027 strain.

| **Gene ID** | **Gene orthologous** | **Name** | **Description** | **Microarray fold change *in vitro*** | **Microarray fold change *in vivo*** |
| --- | --- | --- | --- | --- | --- |
| CDR0833 | CD630_09030 |  | Aldo/keto reductase | 2.51 | 3.24 |
| CDR0971 | CD630_11350 |  | Cell wall hydrolase | 0.44 | 0.44 |
| CDR1823 | CD196_1778 |  | Lipoprotein signal peptidase | 0.53 | 2.52 |
| CDR1914 | CD630_19900 |  | Hypothetical protein | 5.98 | 5.96 |
| CDR0122 | CD630_01230 | *murA* | UDP-N-acetylglucosamine 1-carboxyvinyltransferase | 0.58 | 2.24 |
| CDR0329 | CD630_03240 | *cbiM* | Cobalt transport protein CbiM | 4.51 | 2.41 |
| CDR1824 | CD630_19040 |  | ABC transporter permease | 2.86 | 2.73 |
| CDR2249 | CD630_23620 |  | Fragment of ABC-type transport system, permease (Part 1) | 0.46 | 0.48 |
| CDR2250 | CD630_23630 |  | Hypothetical protein | 0.34 | 0.31 |
| CDR2253 | CD630_23660 |  | Hypothetical protein | 0.34 | 0.29 |
| CDR2254 | CD630_23670 |  | Permease | 0.40 | 0.45 |
| CDR2533 | CD630_26450 |  | Extracellular solute-binding protein | 0.41 | 3.06 |
| CDR2554 | CD630_26660 | *ptsG-A* | PTS system glucose-specific transporter subunit IIA | 0.30 | 0.26 |
| CDR2555 | CD630_26670 | *ptsG-BC* | PTS system, glucose-specific IIBC component | 0.36 | 0.36 |
| CDR3096 | CD630_32360 |  | Hypothetical protein | 4.26 | 2.25 |
| CDR2839 | CD630_30030 |  | Carbohydrate hydrolase (N-terminus) | 0.31 | 0.47 |
| CDR3281 | CD196_3235 |  | transposon Tn21 resolvase | 2.90 | 3.65 |
| CDR0240 | CD630_02390 | *fliC* | Flagellin subunit | 0.14 | 0.11 |
| CDR0252 | CD630_02490 | *fliG* | Flagellar motor switch protein | 0.55 | 0.18 |
| CDR0253 | CD630_02500 | *fliH* | Flagellar assembly protein | 0.43 | 0.35 |
| CDR0592 | CD630_06690 |  | Two-component sensor histidine kinase | 0.44 | 0.21 |
| CDR1105 | CD630_12650 |  | Regulatory protein | 2.45 | 3.49 |
| CDR1127 | CD630_12870 | *fur* | Ferric uptake regulation protein | 0.24 | 2.21 |
| CDR2929 | CD630_30900 | *treR* | GntR family transcriptional regulator | 0.16 | 3.72 |
| CDR3021 | CD630_31670 |  | Phosphatidylethanolamine-binding regulatory protein | 2.85 | 2.38 |
| CDR3278 | CD196_3232 |  | Hypothetical protein | 5.73 | 4.25 |
| CDR3193 | CD630_33490 | *bclA3* | Exosporium glycoprotein | 10.04 | 2.82 |
| CDR3406 | CD630_35690 |  | Peptidase | 2.17 | 2.98 |
| CDR1335 | CD630_14860 |  | Lipoprotein | 3.06 | 4.70 |
| CDR0920 | CD630_10631 |  | Hypothetical protein | 4.17 | 7.57 |
| CDR0969 | CD630_11330 |  | Hypothetical protein | 4.88 | 4.28 |
| CDR1558 | CD630_16600 |  | Hypothetical protein | 0.43 | 2.42 |
| CDR1688 | CD630_17930 |  | hypothetical protein | 2.73 | 2.43 |
| CDR3094 | CD630_32340 |  | hypothetical protein | 2.78 | 2.43 |
| CDR3285 | CD196_3239 |  | hypothetical protein | 4.37 | 5.45 |
| CDR3286 | CD196_3240 |  | hypothetical protein | 3.96 | 3.09 |
